# VIK‐Mediated Auxin Signaling Regulates Lateral Root Development in *Arabidopsis*

**DOI:** 10.1002/advs.202402442

**Published:** 2024-07-03

**Authors:** Erlei Shang, Kaijing Wei, Bingsheng Lv, Xueli Zhang, Xuefeng Lin, Zhihui Ding, Junchen Leng, Huiyu Tian, Zhaojun Ding

**Affiliations:** ^1^ The Key Laboratory of Plant Development and Environmental Adaptation Biology Ministry of Education School of Life Sciences Shandong University Qingdao Shandong 266237 China; ^2^ College of Horticulture Qingdao Agricultural University Qingdao Shandong 266109 China

**Keywords:** auxin, ERF13, lateral root, LBD18, VIK

## Abstract

The crucial role of TIR1‐receptor‐mediated gene transcription regulation in auxin signaling has long been established. In recent years, the significant role of protein phosphorylation modifications in auxin signal transduction has gradually emerged. To further elucidate the significant role of protein phosphorylation modifications in auxin signaling, a phosphoproteomic analysis in conjunction with auxin treatment has identified an auxin activated Mitogen‐activated Protein Kinase Kinase Kinase (MAPKKK) VH1‐INTERACTING Kinase (VIK), which plays an important role in auxin‐induced lateral root (LR) development. In the *vik* mutant, auxin‐induced LR development is significantly attenuated. Further investigations show that VIK interacts separately with the positive regulator of LR development, LATERAL ORGAN BOUNDARIES‐DOMAIN18 (LBD18), and the negative regulator of LR emergence, Ethylene Responsive Factor 13 (ERF13). VIK directly phosphorylates and stabilizes the positive transcription factor LBD18 in LR formation. In the meantime, VIK directly phosphorylates the negative regulator ERF13 at Ser168 and Ser172 sites, causing its degradation and releasing the repression by ERF13 on LR emergence. In summary, VIK‐mediated auxin signaling regulates LR development by enhancing the protein stability of LBD18 and inducing the degradation of ERF13, respectively.

## Graphical abstract

VH1‐INTERACTING Kinase (VIK) plays a crucial positive role in auxin‐triggered protein phosphorylation during lateral root (LR) development. Upon activation, VIK phosphorylates the positive transcription factor LATERAL ORGAN BOUNDARIES‐DOMAIN18 (LBD18) and the negative regulator of LR emergence, Ethylene Responsive Factor 13 (ERF13). This phosphorylation enhances the stability of LBD18 and induces the degradation of ERF13, thereby promoting LR formation.

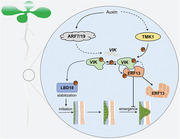

## 1 Introduction

Lateral roots (LRs) are the post‐embryonic organs and represent crucial structures in the root system of terrestrial plants.^[^ ^1^, ^2^, ^3^, ^4^ ^]^ LRs exhibit numerous branches and a broad surface area, providing the foundation for supporting plant growth and development by mediating soil anchorage and the absorption of water and minerals.^[^ ^3^, ^5^, ^6^ ^]^ The organogenesis of LR is a complex developmental process. The pericycle cell at the xylem pole, located within basal meristem zone of the parent root, is specified to the founder cell (FC).^[^ ^7^ ^]^ This cell undergoes asymmetric divisions, initiating LR development and generating LR primordia (LRP).^[^ ^7^, ^8^ ^]^ Subsequently, coordinated cell divisions and mechanical effects from the overlying cell layers act together to promote LRP outgrowth, forming a dome‐like LRP shape.^[^ ^7^, ^9^ ^]^ As the LRP penetrates through the endodermis, cortex, and epidermis, the LR eventually emerges into the rhizosphere, signifying the formation of an emerged lateral root (LRE) and subsequent development into a mature LR.^[^ ^4^ ^]^ And most of the studies have been focused on LR initiation and emergence.^[^ ^10^ ^]^

Auxin is a crucial regulatory factor in LR development, participating in the regulation of almost every stage, including initiation and emergence, among various developmental phases.^[^ ^11^, ^12^, ^13^, ^14^ ^]^ Auxin signaling is transduced through the receptor transport inhibitor response 1 (TIR1)/Auxin Signaling F‐Boxs (AFBs), the transcriptional repressor Auxin/Indole‐3‐Acetic Acids (Aux/IAAs), and the auxin response factors (ARFs).^[^ ^15^, ^16^, ^17^, ^18^, ^19^ ^]^ The IAA28‐ARF7/ARF19 auxin signaling module regulates the priming of LR FCs through the regulation of GATA23 transcription factor.^[^ ^20^, ^21^ ^]^ Following auxin established FC identity, auxin triggers LR initiation through SLR/IAA14‐ARF7/ARF19 and BODENLOS (BDL)/IAA12‐MONOPTEROS (MP)/ARF5 signaling modules.^[^ ^22^, ^23^, ^24^, ^25^ ^]^ The Lateral Organ Boundaries‐Domain/Asymmetric Leaves2‐like (LBD/ASL) factors such as LBD16 and LBD18 act downstream of ARF7/ARF19‐mediated auxin signaling to regulate LR formation at the initiation and emergence steps.^[^ ^26^, ^27^, ^28^, ^29^ ^]^ Cell cycle related genes such as *E2Fa* and cell well remodeling related genes such as *EXPANSIN14* (*EXP14*) are further activated through LBDs transcription factors, thereby regulating the initiation and emergence of auxin‐induced LR development.^[^ ^28^, ^29^, ^30^ ^]^

Since the identification of auxin receptor TIR1, auxin signaling has been known to be transduced through the classical transcriptional regulatory pathways mediated by TIR1/AFBs receptors and the ARFs‐AUX/IAAs regulatory modules, and regulate various plant growth and development.^[^ ^15^, ^31^, ^32^, ^33^ ^]^ In addition to this above classical transcriptional regulatory auxin signaling pathway, accumulating evidences have shown that auxin‐induced protein phosphorylation modifications also play a profoundly important role in auxin signal transduction and the auxin‐mediated plant growth and development.^[^ ^34^, ^35^, ^36^, ^37^, ^38^, ^39^, ^40^, ^41^, ^42^, ^43^, ^44^, ^45^ ^]^ For example, auxin induces cleavage of transmembrane kinase 1 (TMK1), and the C terminus of TMK1 translocates into the nucleus to specifically phosphorylate and stabilize two non‐canonical transcriptional repressors, IAA32 and IAA34, thereby regulating differential growth of the apical hook.^[^ ^35^ ^]^ Auxin activated TMK1 also regulates apoplastic acidification and cell expansion through phosphorylating plasma membrane H+‐ATPase.^[^ ^37^ ^]^ During LR formation, auxin regulates cell division pattern in LRP through Mitogen‐activated Protein Kinase Kinase 4/5 (MKK4/5)‐MPK3/6 signaling cascade.^[^ ^36^ ^]^ Auxin‐activated MPK14 has been found to regulate LR emergence through phosphorylation and degradation of Ethylene Responsive Factor 13 (ERF13), a negative regulator of LR emergence.^[^ ^40^, ^46^ ^]^

To further elucidate the significant role of protein phosphorylation in the transmission of auxin signaling and identify pivotal regulatory factors involving protein phosphorylation during auxin‐induced LR formation, we conducted phosphoproteomic analysis using 10‐day‐old *Arabidopsis* seedling roots, with 10 µM 1‐Naphthylacetic acid (NAA) treatment for 15 min and 8 h, respectively. Through this analysis, we identified that VH1‐INTERACTING Kinase (VIK), a member of C1 subfamily Mitogen‐activated Protein Kinase Kinase Kinase (MAPKKK), is activated by auxin and plays a positive regulatory role in LR development. We found that VIK interacts respectively with the positive regulator of LR development, LBD18, and the negative regulator of LR emergence, ERF13. During LR formation, VIK directly phosphorylates and stabilizes the transcription factor LBD18. In the process of LR emergence, VIK phosphorylates ERF13 at Ser168 and Ser172, leading to its degradation, thereby promoting LR emergence.

## 2 Results

### 2.1 Auxin Promotes VIK Phosphorylation and its Transcription

**Figure 1:**
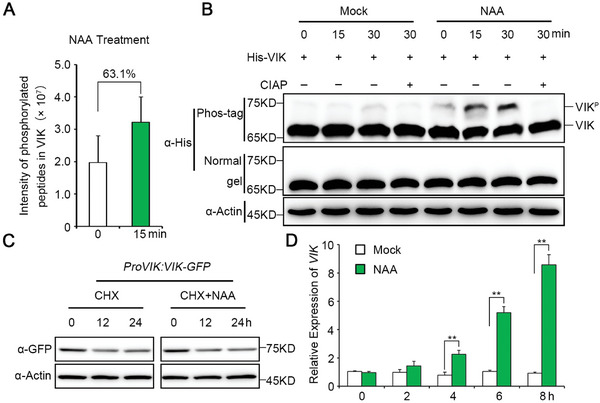
Auxin promotes VIK phosphorylation and its transcription. A) The phosphopeptides intensity of VIK from root total protein of 10‐day‐old wild‐type (WT) seedlings with or without NAA treatment (10 µM NAA for 15 min). B) VIK phosphorylation in a cell‐free assay. The total proteins were extracted from WT seedlings treated with or without 10 µM NAA, and then mixed with His‐VIK recombinant protein to incubate for 0, 15, and 30 min respectively. His‐VIK was separated by the Phos‐tag SDS‐PAGE gel and detected by anti‐His antibody. The phosphorylated VIK was dramatically inhibited by calf intestinal alkaline phosphatase (CIAP). Actin served as a loading control. C) Ten‐day‐old *ProVIK:VIK‐GFP* seedlings were treated with 200 µM cycloheximide (CHX) or 200 µM CHX + 10 µM NAA for 12 and 24 h, respectively. VIK was detected by anti‐GFP antibody. Actin served as a loading control. D) *VIK* mRNA levels in roots of 10‐day‐old WT seedlings were assessed at various time points following treatment with 10 µM NAA. The expression levels were quantified by quantitative real‐time PCR (qRT‐PCR) with *ACTIN2* served as an internal control. Error bars indicate SD of three biological replicates. Asterisks represent significant differences compared to WT used Student's *t* test (^**^ *P* < 0.01).

To identify potential phosphorylated regulatory factors triggered by auxin, roots of 10‐day‐old *Arabidopsis* seedlings treated with 10 µM NAA for 15 min and 8 h, respectively, were collected to perform protein phosphoproteome analysis. We obtained 5309 phosphorylated peptides containing 11 577 phosphorylation sites that are distributed in 2058 proteins (Figure S1A and Table S1, Supporting Information). Among the pool of phosphorylated proteins, a total of 155 protein kinases were identified, with 22 belonging to the MAPK signaling cascades (Figure S1B and Table S1, Supporting Information). Notably, VH1‐INTERACTING Kinase (VIK), also known as Integrin‐Linked Kinase 6 (ILK6),^[^ ^47^, ^48^ ^]^ and a member of the MAPKKK C1‐subfamily,^[^ ^49^ ^]^ exhibited a substantial 63.1% increase in the abundance of phosphorylated peptides after 15 min of NAA treatment compared to the untreated control (**Figure** **1**A; Table S1, Supporting Information). This finding strongly suggests that VIK may play a pivotal role in mediating auxin signaling through phosphorylation modifications.

To further verify the above results, His‐VIK recombinant protein, comprising an N‐terminal 6 × histidine (His)‐tag and a full‐length VIK protein, was expressed in *Escherichia coli*, and the purified His‐VIK was used to carry out phosphorylation assays in a cell‐free protein system. Recombinant His‐VIK was incubated with extracted total protein from roots of 10‐day‐old *Arabidopsis* seedlings. We found that His‐VIK exhibits basal phosphorylation at the beginning (Figure 1B), with a gradual and slight increase in its phosphorylation level over time (Figure 1B). As expected, we found that the phosphorylation level of His‐VIK was significantly increased in root tissue lysate of wild‐type (WT) seedlings treated with NAA within 15 and 30 min (Figure 1B), suggesting that auxin can significantly promote VIK phosphorylation. Given that TMK1 is a crucial kinase activated by auxin and mediates auxin‐triggered protein phosphorylation, we investigated the potential interaction between VIK and TMK1. Using yeast two‐hybrid (Y2H) analysis, we identified a direct interaction between them (Figure S2A, Supporting Information). This interaction was also confirmed by luciferase complementation imaging (LCI) assays in *Nicotiana benthamiana* leaves (Figure S2B, Supporting Information). To further determine whether TMK1 can phosphorylate VIK, we purified recombinant proteins His‐VIK and TMK1‐GFP, and performed in vitro phosphorylation assays using anti‐thiophosphate ester antibody (anti‐TPE). The results showed that VIK was phosphorylated by TMK1 (Figure S2C, Supporting Information).

We also investigated whether auxin has an effect on the protein stability of VIK by generating a *ProVIK:VIK‐GFP* transgenic line. The protein degradation assays in vivo showed that auxin may not affect the VIK protein stability (Figure 1C). To further investigate whether auxin affects *VIK* transcription, we performed a time‐course quantitative real‐time PCR (qRT‐PCR) analysis to monitor *VIK* mRNA levels in roots of WT seedlings treated with NAA. Our findings revealed a time‐dependent induction of *VIK* transcript levels in response to auxin (Figure 1D). Further analysis reveals that the induction of *VIK* expression is primarily mediated in an ARF7/19‐dependent manner. This is evidenced by a notably diminished induction of *VIK* expression in the *arf7/19* mutant, contrasting with the *lbd16/18/33* mutant where *VIK* expression remains significantly induced by auxin (Figure S3, Supporting Information).

These findings imply that auxin plays a role in promoting both the transcription of *VIK* and its subsequent phosphorylation modifications.

### 2.2 VIK‐Mediated Auxin Signaling Positively Regulates LR Development

**Figure 2:**
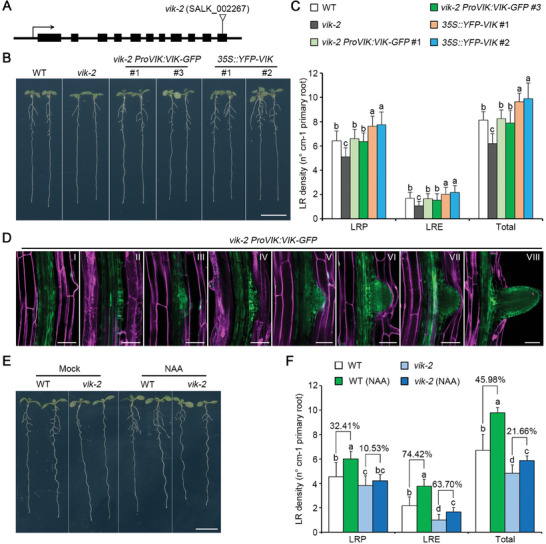
VIK‐mediated auxin signaling positively regulates LR development. A) Schematic diagram illustrating the T‐DNA insertion site of the *vik‐2* mutant in *VIK* locus. B) LR phenotypes were examined in 10‐day‐old seedlings, including WT, *vik‐2*, two distinct rescued lines (*vik‐2 ProVIK:VIK‐GFP*), and two independent overexpression lines (*35S::YFP‐VIK*). Scale bar, 1 cm. C) LR density of WT, *vik‐2*, *vik‐2 ProVIK:VIK‐GFP*, and *35S::YFP‐VIK* seedlings. LR density refers to the ratio of the number of LR to primary root length. LRP, LRE and Total represent LR primordia, emerged LR and total LR, respectively. Data are indicated as means ± SD (*n* = 30). D) Expression patterns of VIK in LRs of *vik‐2 ProVIK:VIK‐GFP*. Green and purple represent GFP signal and PI staining cell boundary, respectively. The roman numeral indicates developmental stage of LR. Scale bars, 40 µm. E) The LR phenotypes were evaluated in 10‐day‐old seedlings of both WT and *vik‐2*, grown on 1/2 MS medium or medium supplemented with 25 nM NAA. Scale bar, 1 cm. F) LR density of seedlings in (E). Data are indicated as means ± SD (*n* = 30). Different letters indicate significant differences in a subset used one‐way ANOVA (*P* < 0.05) in both (C) and (F).

Auxin has been known to play a critical role in promoting LR formation. To examine whether VIK is involved in auxin‐induced LR development, two T‐DNA insertion mutants, denoted as *vik‐2* and *vik‐3* (**Figure** **2**A; Figure S4A,B, Supporting Information), were identified for further analysis of LR phenotypes. Compared to WT, both *vik‐2* and *vik‐3* mutant seedlings exhibited a significant decrease in total LR density, along with LR primordia (LRP) and emerged LR (LRE) (Figure 2B,C; Figure S4C,D, Supporting Information), with *vik‐2* exhibiting a particularly noteworthy decrease (Figure 2B,C). Conversely, two independent *35S::YFP‐VIK* transgenic lines showed increased LRs density (Figure 2B,C). Additionally, we generated *ProVIK:VIK‐GFP* transgenic lines in the *vik‐2* mutant background and observed the successful restoration of the defective LR density phenotype of *vik‐2* (Figure 2B,C). To further evaluate the impact of VIK on LR development, we applied a gravistimulation to synchronize LR formation and counted LR developmental stages at 18 and 48 h after induction.^[^ ^7^ ^]^ The results showed that, compared to the WT control, the *vik‐2* mutant seedlings exhibit a noticeable deceleration in LR development, whereas *35S::YFP‐VIK* seedlings demonstrate a significant acceleration in LR development (Figure S4E, Supporting Information).

Next, we investigated the expression patterns of VIK by creating a transgenic reporter line, *ProVIK:VIK‐GUS*, which expresses the chimeric VIK‐GUS protein. The results of GUS staining revealed robust expression of VIK not only in leaf veination, the hypocotyl, primary root tip, and stele (Figure S5A–C, Supporting Information), but also in LRs (Figure S5D,E, Supporting Information). Consistent with above qRT‐PCR results (Figure 1D), we observed enhanced GUS signals in both LRP and LRE of *ProVIK:VIK‐GUS* plants treated with NAA for 12 h compared to the mock treatment (Figure S5D,E, Supporting Information). Moreover, using *vik‐2 ProVIK:VIK‐GFP* transgenic lines, we consistently detected GFP signals in all LRs (Figure 2D), as well as in the cells of endodermis, cortex and epidermis of primary root (Figure 2D; Figure S5F, Supporting Information). Additionally, the VIK protein exhibited a nucleocytoplasmic distribution, which was confirmed by the colocalization of YFP‐VIK and BZR1‐RFP, a marker for nucleocytoplasmic localization,^[^ ^50^ ^]^ in the leaf cells of *N. benthamiana* (Figure S6A, Supporting Information). In *vik‐2 ProVIK:VIK‐GFP* lines, a consistent subcellular localization pattern was also observed in LR cells (Figure S6B,C, Supporting Information).

To explore the roles of VIK in auxin‐induced LR development, we examined the LR density in both WT and the *vik‐2* mutant seedlings combined with NAA treatment. The findings revealed that the increase in LR density induced by auxin in the WT was notably reduced in the *vik‐2* mutant (Figure 2E,F). This suggests a critical role for VIK in the auxin‐induced regulation of LR development. In summary, these results highlight VIK as a crucial mediator through which auxin positively regulates LR development.

### 2.3 VIK Physically Interacts with ERF13

**Figure 3:**
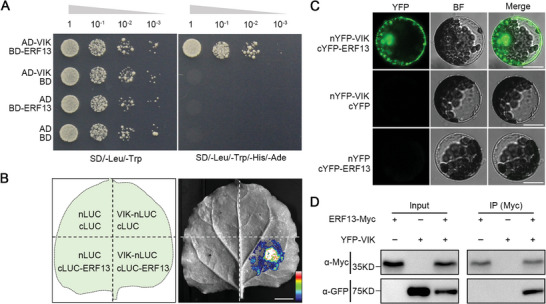
VIK interacts with ERF13. A) Yeast Two‐Hybrid (Y2H) assay. VIK was utilized as the prey in the yeast two‐hybrid system by fusing it to the C‐terminal of the activation domain (AD). ERF13 served as the bait by fusing it to the C‐terminal of binding domain (BD). Yeast cells were grown on synthetic dextrose minimal medium without leucine and tryptophan (SD/‐Leu/‐Trp) and synthetic dextrose minimal medium without leucine, tryptophan, histidine and adenine (SD/‐Leu/‐Trp/‐His/‐Ade) supplemented with 25 mM 3‐amino‐1,2,4‐triazole (3‐AT). AD or BD represents empty vector. B) Luciferase Complementation Imaging (LCI) assay. The full‐length VIK and ERF13 were fused individually with nLUC and cLUC to create VIK‐nLUC and cLUC‐ERF13, respectively. The built‐up vectors were co‐transformed into leaves of *Nicotiana benthamiana*. The color column indicates the range of luminescence intensity. Scale bar, 1 cm. C) Bimolecular Fluorescence Complementation (BiFC) analysis. The full‐length VIK and ERF13 were individually fused to nYFP and cYFP, generating nYFP‐VIK and cYFP‐ERF13, respectively. The combinations of vectors were co‐transformed into *Arabidopsis* protoplasts. Green represents YFP signal. BF represents bright field. Scale bar, 50 µm. D) Co‐immunoprecipitation (co‐IP) assay. In *Arabidopsis* protoplasts, ERF13‐Myc and YFP‐VIK proteins were expressed individually as well as in combination. The total protein (input) was immunoprecipitated with anti‐Myc beads. VIK was detected by anti‐GFP antibody, and ERF13 was detected by anti‐Myc antibody.

ERF13 has been shown to mediate auxin signaling in LR emergence.^[^ ^40^, ^46^ ^]^ In this study, our phenotype analysis highlights a crucial role of VIK in auxin‐induced LR development (Figure 2). Therefore, we hypothesized that ERF13 could be a potential substrate of VIK. To explore this hypothesis, we initially utilized Y2H analysis to examine whether there is an interaction between VIK and ERF13, and found that VIK directly interacts with ERF13 (**Figure** **3**A). The interaction between VIK and ERF13 was also confirmed by LCI assays in *N. benthamiana* leaves and by bimolecular fluorescence complementation (BiFC) assays in *Arabidopsis* protoplasts (Figure 3B,C). Additionally, we performed a coimmunoprecipitation (co‐IP) assay using *Arabidopsis* protoplasts co‐expressing *35S::ERF13‐Myc* and *35S::YFP‐VIK* constructs. The results demonstrated the coimmunoprecipitation of YFP‐VIK with ERF13‐Myc (Figure 3D). Taken together, these results indicated that VIK physically interacts with ERF13.

### 2.4 VIK Regulates ERF13 Phosphorylation in a Positive Feedback Manner

**Figure 4:**
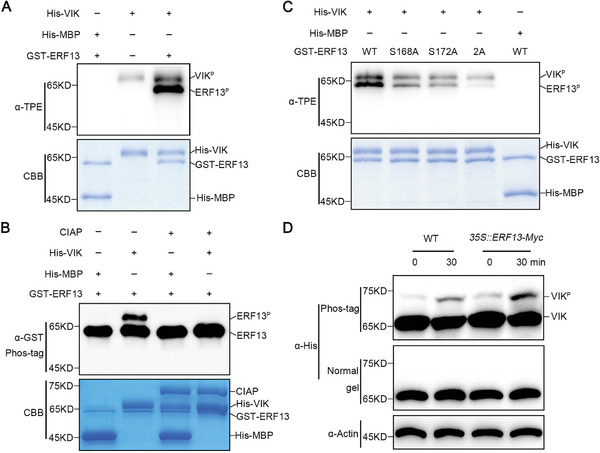
VIK phosphorylates ERF13 at Ser168 and Ser172 sites in a positive feedback manner. A) In the in vitro phosphorylation assay, an anti‐thiophosphate ester (TPE) antibody was employed. The reaction system composed of recombinant GST‐ERF13, His‐VIK, ATP^γ^, and reaction buffer. B) Phosphorylated ERF13 was visualized using anti‐GST antibody after separation by the Phos‐tag SDS‐PAGE gel. The up‐shift band indicates the presence of phosphorylated ERF13. C) The candidate phosphorylation sites in ERF13 were confirmed using an in vitro phosphorylation assay. Recombinant GST‐ERF13, along with its mutant variants GST‐ERF13^S168A^, GST‐ERF13^S172A^, and GST‐ERF13^2A^, were incubated with His‐VIK in an in vitro reaction buffer. All of recombinant proteins were indicated by Coomassie brilliant blue (CBB) staining in (A–C). His‐MBP used as a negative control. D) VIK phosphorylation in a cell‐free assay. The total proteins were extracted from WT and *35S:ERF13‐Myc* seedlings, and then mixed with His‐VIK recombinant protein to incubate for 0 and 30 min, respectively. Phosphorylated VIK was separated by the Phos‐tag SDS‐PAGE gel and detected using an anti‐His antibody. The up‐shift band indicates the presence of phosphorylated VIK. Actin served as a loading control.

The detected interaction between VIK and ERF13 suggests that VIK has the capability to phosphorylate ERF13. Furthermore, our phosphoproteomics analysis revealed that ERF13 phosphorylation was rapidly induced by NAA treatment (Figure S7 and Table S1, Supporting Information). To examine this possibility, we purified recombinant proteins His‐VIK and GST‐ERF13, and performed in vitro phosphorylation assays using anti‐TPE. The results showed that ERF13 was phosphorylated by VIK, which exhibits autophosphorylation activity (**Figure** **4**A). To validate this observation, the phosphorylation status of ERF13 was assessed through in vitro Phos‐tag SDS‐PAGE gel analysis. It was observed that GST‐ERF13 exhibited a distinct upshift band when incubated with His‐VIK (Figure 4B). Furthermore, treatment with calf intestinal alkaline phosphatase (CIAP) resulted in the disappearance of the observed upshift band (Figure 4B), further confirming that VIK can directly phosphorylate ERF13.

To further determine the ERF13 functional phosphorylation site(s) recognized by VIK, we conducted phosphorylation mass spectrometry assay. Two potential sites, Ser168 and Ser172, were identified within ERF13 during in vitro phosphorylation reaction (Figure S8A,B, Supporting Information). Mutation of Ser168 or Ser172 site to the non‐phosphorylated form residue Ala (ERF13^S168A^ or ERF13^S172A^) can moderately weaken the phosphorylation of ERF13 (Figure 4C). Furthermore, simultaneous mutation of these two sites to Ala (ERF13^2A^) nearly abolished the phosphorylation of ERF13 by VIK (Figure 4C), indicating that both Ser168 and Ser172 in ERF13 are major sites phosphorylated by VIK.

The above results also indicated that ERF13 can significantly enhance the autophosphorylation of VIK (Figure 4A,C), and this effect was weakened by non‐phosphorylated ERF13 (Figure 4C). Given that kinase autophosphorylation is an effectively indicator of its kinase activity,^[^ ^51^ ^]^ these findings strongly imply that ERF13, in turn, potentially enhances VIK kinase activity in a substrate phosphorylation‐dependent manner. To corroborate this hypothesis, we conducted additional in vitro phosphorylation assays employing a constitutively active ERF13^S168D^, Ser168 site was substituted with phosphomimic aspartic acid (D), as substrate. The results showed that VIK exhibited a stronger autophosphorylation incubated with ERF13^S168D^ compared to ERF13^WT^ or ERF13^S168A^ (Figure S9, Supporting Information). In addition, we also found that VIK phosphorylation was promoted by ERF13 in a cell‐free phosphorylation assay. VIK displayed a much stronger phosphorylation level incubated with *35S::ERF13‐Myc* seedlings extracts compared to the WT control (Figure 4D). In summary, these results suggested that VIK phosphorylates ERF13 in a positive feedback manner.

Since autophosphorylation of kinases in both animals and plants often requires dimerization,^[^ ^52^, ^53^, ^54^ ^]^ we next investigated whether VIK undergoes homologous dimerization in vivo. The interaction between VIK‐VIK was detected in both Y2H and LCl assays, indicating that VIK can form homologous dimers (Figure S10A,B, Supporting Information). Subsequent in vitro phosphorylation assays revealed that His‐VIK was directly phosphorylated by GST‐VIK, in contrast to the GST‐tag protein control (Figure S10C, Supporting Information), suggesting that VIK is an autophosphorylation kinase. To test whether phosphorylated ERF13 enhances VIK autophosphorylation by promoting its homodimerizations, we conducted LCI experiments and found that the formation of VIK‐VIK dimers was not distinctly influenced by different mutant forms of ERF13 (Figure S10D, Supporting Information).

In summary, these results indicate that VIK positively regulates the phosphorylation of ERF13 by recognizing the Ser168 and Ser172 sites through a feedback mechanism.

### 2.5 VIK‐Mediated Phosphorylation Promotes ERF13 Degradation

**Figure 5:**
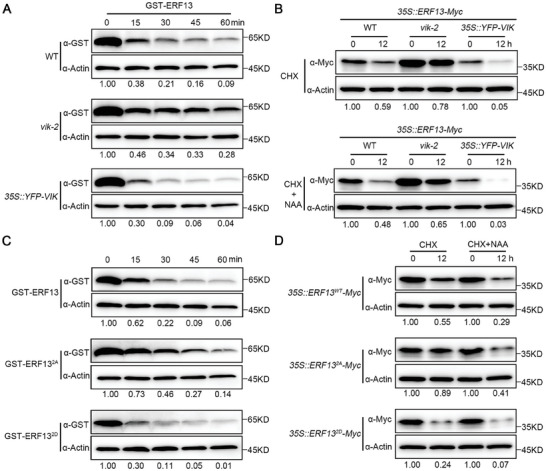
VIK‐mediated phosphorylation accelerates ERF13 degradation. A) Cell‐free assays showed that VIK promotes ERF13 protein degradation in vitro. Total proteins were extracted from 10‐day‐old seedlings of WT, *vik‐2*, and *35S::YFP‐VIK*, and incubated with recombinant GST‐ERF13 for 0, 15, 30, 45, and 60 min, respectively. GST‐ERF13 protein was detected by anti‐GST antibody. Actin served as a loading control. B) ERF13 stability was reduced by VIK in vivo. Ten‐day‐old seedlings of *35S::ERF13‐Myc*, *vik‐2 35S::ERF13‐Myc*, and 3*5S::YFP‐VIK 35S::ERF13*‐*Myc* were subjected to treatments with 200 µM CHX or a combination of 200 µM CHX + 10 µM NAA for 0 and 12 h, respectively. ERF13 was detected by anti‐Myc antibody. Actin served as a loading control. C) Cell‐free assay showing VIK‐mediated ERF13 phosphorylation promotes ERF13 degradation. The total proteins were extracted from 10‐day‐old WT seedlings and incubated with recombinant GST‐ERF13, GST‐ERF13^2A^, and GST‐ERF13^2D^ for 0, 15, 30, 45, and 60 min, respectively. GST‐ERF13 protein was detected by anti‐GST antibody. Actin served as a loading control. D) VIK‐mediated phosphorylation affects ERF13 protein stability in vivo. Ten‐day‐old seedlings of *35S::ERF13^WT^‐Myc*, *35S::ERF13^2A^‐Myc* and *35S::ERF13^2D^‐Myc* were subjected to treatments with 200 µM CHX or a combination of 200 µM CHX + 10 µM NAA for 0 and 12 h, respectively. ERF13 was detected by anti‐Myc antibody. Actin served as a loading control. The abundance of ERF13 relative to Actin was calibrated to 1.00 at 0 h in (A‐D).

Using *ProERF13:ERF13‐YFP* transgenic lines, we observed that ERF13 protein was only expressed in the early LRPs, and became undetectable from stage IV onward (Figure S11A, Supporting Information). In contrast, the VIK protein was continuously expressed in all LRs (Figure S11B, Supporting Information). To investigate whether VIK‐mediated phosphorylation affects the protein stability of ERF13, we examined ERF13 protein stability by performing cell‐free assays. The results showed that the degradation rates of GST‐ERF13 were delayed incubated with total protein from *vik‐2* seedlings compared to WT (**Figure** **5**A), whereas GST‐ERF13 was more unstable in *35S::YFP‐VIK* seedling lysate (Figure 5A). Furthermore, we investigated whether ERF13 protein stability was affected by VIK in vivo and found that, compared to the *35S::ERF13‐Myc* control, the degradation of ERF13 after cycloheximide (CHX) treatment was further accelerated in *35S::YFP‐VIK* background, whereas it exhibited a delayed degradation in the *vik* mutant (Figure 5B). We also found that, in comparison with auxin‐promoted ERF13 degradation in the WT background after NAA treatment, this degradation was attenuated in the *vik* mutant and enhanced in the *35S::ERF13‐Myc* line (Figure 5B). All these results indicate that VIK‐mediated auxin signaling promotes ERF13 degradation.

Next, we generated a phosphomimic ERF13 variant whereby the Ser168 and Ser172 site residues were mutated to aspartic acid (D) in ERF13^2D^ and a phosphor inactive version whereby the Ser168 and Ser172 site residues were mutated to alanine (A) in ERF13^2A^. We then measured the degradation rates of recombinant GST‐ERF13^2A^, GST‐ERF13, and GST‐ERF13^2D^ incubated with total protein extracts from WT seedlings in a cell‐free system. Recombinant GST‐ERF13^2D^ appeared to be degraded much faster than GST‐ERF13, whereas GST‐ERF13^2A^ was more stable (Figure 5C), suggesting that phosphorylation destabilizes ERF13. To further test phosphorylation at Ser168 and Ser172 sites on ERF13 protein stability in vivo, we compared the protein stability among the WT version of ERF13, phosphor inactive version of ERF13^2A^, and phosphomimic version of ERF13^2D^, using *35S::ERF13^WT^‐Myc, 35S::ERF13^2A^‐Myc* and *35S::ERF13^2D^‐Myc* transgenic plants. We selected these transgenic lines possessing comparable transcript level of *ERF13* (Figure S12, Supporting Information) to examine ERF13 protein degradation combined with CHX or NAA treatment. In the presence of CHX, the protein level of ERF13 was greatly reduced in *35S::ERF13^WT^‐Myc* transgenic plants but was only moderately reduced in *35S::ERF13^2A^‐Myc* transgenic plants. However, as shown in *35S::ERF13^2D^‐Myc* transgenic plants, the degradation of ERF13^2D^ was accelerated compared with the ERF13^WT^ (Figure 5D). Next, we examined the phosphorylation effect on ERF13 protein stability with auxin treatment. Obviously, auxin‐promoted degradation of ERF13 was slowed down in *35S::ERF13^2A^‐Myc*, whereas the same auxin treatment significantly enhanced the degradation rate of ERF13 in *35S::ERF13^2D^‐Myc* (Figure 5D).

Similar to VIK‐mediated auxin signaling promotes ERF13 degradation, MPK14‐mediated phosphorylation was also shown to trigger ERF13 degradation.^[^ ^40^ ^]^ To investigate the relationship between VIK and MPK14 in regulating the stability of ERF13 protein, we first examined whether VIK interacts with MPK14 using a Y2H analysis, but no such interaction was observed (Figure S13A, Supporting Information). Subsequently, we engineered various recombinant ERF13 variants in *E. coli*, including GST‐ERF13^WT^, GST‐ERF13^2D^ (the Ser168 and Ser172 sites were substituted with D), GST‐ERF13^3D^ (the Thr66, Ser67, and Thr124 sites recognized by MPK14 were substituted with D), and GST‐ERF13^5D^ (all the five sites above‐mentioned were substituted with D), and measured the degradation rates of these recombinants by performing cell‐free assays. The results showed that all mutant versions of ERF13 exhibited faster degradation compared to WT (Figure S13B, Supporting Information), with GST‐ERF13^5D^ exhibiting the fastest degradation rates (Figure S13B, Supporting Information). These findings suggest a collaborative role of VIK‐ and MPK14‐mediated phosphorylation in promoting ERF13 degradation.

We also examined whether VIK‐mediated phosphorylation affects the subcellular localization of ERF13 proteins using *35S::ERF13‐GFP*, *35S::ERF13^2A^‐GFP*, and *35S::ERF13^2D^‐GFP* transgenic seedlings, and found that both phosphomimic variant ERF13^2D^ and phosphor inactive variant ERF13^2A^ were located in the nucleus, exhibiting no discernible differences from the WT version of ERF13 (Figure S14, Supporting Information). In addition, the qRT‐PCR assays showed that there was no significant change of *ERF13* mRNA level in *vik‐2* mutant compared to the WT (Figure S15, Supporting Information), while the mRNA levels of its downstream regulated‐genes, *KCS8* and *KCS16*, were significantly decreased (Figure S15, Supporting Information), further indicating that VIK regulates ERF13 at the protein level.

In summary, these results indicated that VIK‐mediated phosphorylation at Ser168 and Ser172 sites of ERF13 negatively regulates its protein stability, which accelerates auxin‐promoted ERF13 degradation.

### 2.6 VIK‐Mediated Phosphorylation of ERF13 Regulates LR Emergence

**Figure 6:**
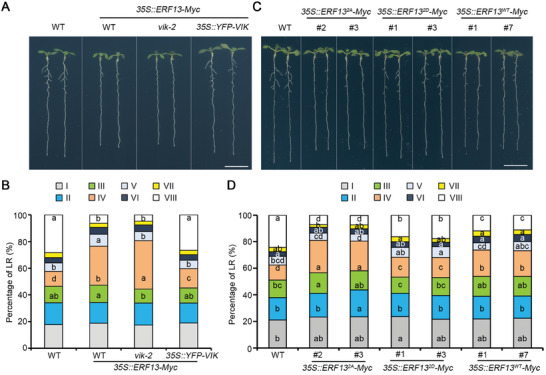
VIK‐mediated phosphorylation of ERF13 promotes LR emergence. A) LR phenotypes were assessed in 10‐day‐old seedlings of WT, *35S::ERF13‐Myc*, *vik‐2 35S::ERF13‐Myc* and *35S::YFP‐VIK 35S::ERF13‐Myc*. Scale bar, 1 cm. B) The proportion of LR at different stages were analyzed in 10‐day‐old seedlings of WT, *35S::ERF13‐Myc*, *vik‐2 35S::ERF13‐Myc* and *35S::YFP‐VIK 35S::ERF13‐Myc*. Roman numerals I‐VIII represent LR developmental stages. C) LR phenotypes were assessed in 10‐day‐old seedlings of WT, *35S::ERF13^2A^‐Myc*, *35S::ERF13^2D^‐Myc* and *35S::ERF13^WT^‐Myc*. Scale bar, 1 cm. D) The proportion of LRs at different stages were analyzed in 10‐day‐old seedlings of WT, *35S::ERF13^2A^‐Myc*, *35S::ERF13^2D^‐Myc* and *35S::ERF13^WT^‐Myc*. Roman numerals I‐VIII represent LR developmental stages. Different letters indicate significant differences of the same developmental stage LR proportion among different plants used one‐way ANOVA (*P* < 0.05, *n* = 40) in both (B) and (D).

Since VIK positively regulates LR development while ERF13 plays a negative role,^[^ ^40^ ^]^ and VIK mediates auxin signaling to enhance ERF13 degradation (Figure 5), we next explored the potential involvement of VIK‐regulated ERF13 degradation in LR development. We compared LR phenotypes using *35S::ERF13‐Myc*, *vik‐2 35S::ERF13‐Myc*, and *35S::YFP‐VIK 35S::ERF13‐Myc* transgenic lines. In line with previous findings,^[^ ^40^ ^]^ we found that LR emergence was highly repressed when ERF13 is overexpressed (**Figure** **6**A; Figure S16A, Supporting Information), and a distinct defect with a delay of LRP development from stage IV to V was observed in *35S::ERF13‐Myc* lines compared to WT (Figure 6B). However, the inhibitory effect of overexpression of ERF13 on LR emergence was enhanced in *vik* and alleviated in the *35S::YFP‐VIK* background respectively (Figure 6A; Figure S16A, Supporting Information). Compared to *35S::ERF13‐Myc* seedlings, the *vik‐2 35S::ERF13‐Myc* seedlings had slightly less emerged LRs and more LRP confined to stage IV, while *35S::YFP‐VIK 35S::ERF13‐Myc* seedlings exhibited almost no repressed LR emergence and WT‐like developmental progression of LRP from stage IV to V (Figure 6A,B; Figure S16A, Supporting Information).

We next compared LR phenotypes using *35S::ERF13^WT^‐Myc*, *35S::ERF13^2A^‐Myc* and *35S::ERF13^2D^‐Myc* transgenic lines. We noted that, in comparison to the WT, all three variants of ERF13‐overexpressing lines showed a decrease in LR emergence and an increase in LRPs (Figure 6C; Figure S16B, Supporting Information). However, compared to the *35S::ERF13^WT^‐Myc* seedlings, *35S::ERF13^2A^‐Myc* seedlings exhibited even less emerged LRs, while *35S::ERF13^2D^‐Myc* transgenic seedlings had a bit more emerged LRs (Figure S16B, Supporting Information). Further analysis revealed that the proportion of LRPs at stages IV was highest (approximately 23%) in *35S::ERF13^2A^‐Myc* seedlings, while around 18% of LRPs were at stage IV in *35S::ERF13^WT^‐Myc* seedlings, and the lowest proportion (approximately 14%) of LRPs at stage IV was observed in *35S::ERF13^2D^‐Myc* seedlings (Figure 6D).

Altogether, these results indicated that VIK‐mediated phosphorylation and degradation of ERF13 promote LR emergence.

### 2.7 VIK Phosphorylates and Stabilizes LBD18 Protein in LR Development

Our above results showed that VIK plays a critical role in various stages of LR development (Figure 2B,C; Figure S4C–E, Supporting Information). To further elucidate the molecular mechanisms underlying VIK regulated LR development, we examined the transcription levels of pivotal genes in LR development, including *GATA23*, *ARF7*, *ARF19*, *LBD16*, *LBD18*, *LBD29*, *LBD33*, and *E2Fa* in the root of *vik‐2* mutant seedlings by qRT‐PCR analysis.^[^ ^8^, ^21^, ^26^, ^30^, ^55^ ^]^ We observed a significant reduction in the mRNA levels of *E2Fa*, a target gene of LBD18,^[^ ^30^ ^]^ in the *vik* mutant compared to the WT (Figure S17, Supporting Information). Furthermore, the transcript level of *EXP14*, another direct target of LBD18,^[^ ^29^ ^]^ was also down‐regulated in *vik‐2* (Figure S17, Supporting Information). These results implied that VIK may influence LBD18 activity.

**Figure 7:**
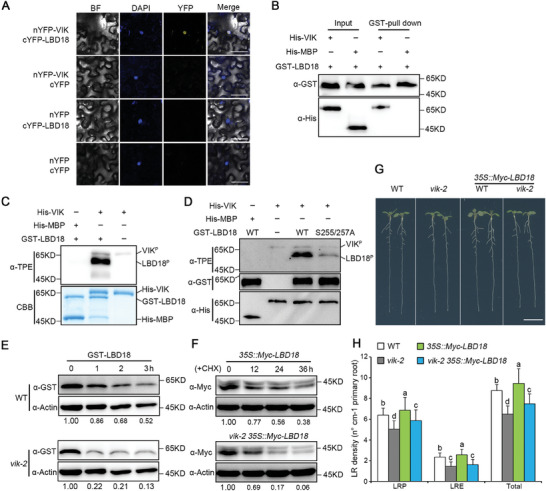
VIK directly phosphorylates and stabilizes LBD18 protein during LR formation. A) BiFC assay. The full‐length VIK and LBD18 were fused individually to nYFP and cYFP to create nYFP‐VIK and cYFP‐LBD18, respectively. The combinations of vectors were co‐transformed into leaf cells of *N. benthamiana*. Yellow and blue indicate YFP and 4′,6‐diamidino‐2‐phenylindole (DAPI) signals, respectively. BF represents bright field. Scale bar, 50 µm. B) In vitro pull‐down analysis. Recombinant GST‐LBD18 was attached to glutathione beads, and incubated with His‐VIK or His‐MBP, respectively. LBD18 was detected by anti‐GST antibody. His‐tag proteins were detected by anti‐His antibody. His‐MBP served as a negative control. C) The in vitro phosphorylation assay. The reaction system composed of recombinant GST‐LBD18, His‐VIK, ATP^γ^ and reaction buffer. Phosphorylated proteins were visualized by anti‐TPE antibody. Loading proteins were detected by CBB staining. D) The candidate phosphorylation sites in LBD18 were confirmed using an in vitro phosphorylation assay. Recombinant GST‐LBD18, along with its mutant variant GST‐LBD18^S255A/257A^ were incubated with His‐VIK in an in vitro reaction buffer. LBD18 was detected by anti‐GST antibody. His‐tag proteins were detected by anti‐His antibody. His‐MBP used as a negative control. E) VIK stabilizes LBD18 protein in vitro by a cell‐free assay. The total proteins were extracted from 10‐day‐old seedlings of WT and *vik‐2*, and subsequent incubated with recombinant GST‐LBD18 for 0, 1, 2, and 3 h, respectively. GST‐LBD18 protein was detected by anti‐GST antibody. Actin served as loading control. F) The protein stability analysis of LBD18 in vivo. Ten‐day‐old seedlings of *35S::Myc‐LBD18* and *vik‐2 35S::Myc‐LBD18* were treated with 200 µM CHX for 0, 12, 24, and 36 h, respectively. LBD18 was detected by anti‐Myc antibody. Actin served as a loading control. The abundance of LBD18 relative to Actin was calibrated to 1.00 at 0 h in (E) and (F). G) LR phenotypes were assessed in 10‐day‐old seedlings of WT, *vik‐2*, *35S::Myc‐LBD18*, and *vik‐2 35S::Myc‐LBD18*. Scale bar, 1 cm. H) LR density of 10‐day‐old WT, *vik‐2*, *35S::Myc‐LBD18* and *vik‐2 35S::Myc‐LBD18* seedlings. LRP, LRE, and Total represent LR primordia, emerged LR and total LR, respectively. LR density refers to the ratio of the number of LR to primary root length. Data are indicated as means ± SD (*n* = 35). Different letters indicate significant differences in a subset using one‐way ANOVA (*P* < 0.05).

Given the findings indicating ubiquitous expression of both LBD18 and VIK proteins across all LRs and the overlaying layer cells of the primary root (Figure S18, Supporting Information), we investigated whether VIK directly interacts with LBD18. We firstly conducted BiFC assay in *N. benthamiana* leaves and observed a clearly YFP signal (**Figure** **7**A), indicating that VIK interacts with LBD18 in vivo. The interaction between VIK and LBD18 was further verified using pull‐down assays (Figure 7B). These results suggested that VIK directly interacts with LBD18.

Our phosphorylomics results also showed that LBD18 phosphorylation was induced by NAA treatment (Figure S19 and Table S1, Supporting Information). To test whether LBD18 acts as a substrate for VIK kinase, purified recombinant proteins GST‐LBD18 and His‐VIK were utilized in in vitro phosphorylation assays. The outcomes demonstrated direct phosphorylation of LBD18 by His‐VIK (Figure 7C). Furthermore, we conducted a phosphorylation mass spectrometry assay to determine the LBD18 functional phosphorylation site(s) recognized by VIK. This analysis revealed that two potential sites, Ser255 and Ser257, within LBD18 were phosphorylated in the in vitro reaction (Figure S20, Supporting Information). Subsequent mutation of these sites to Ala (S255/257A) significantly attenuated the phosphorylation of LBD18 by VIK (Figure 7D), indicating their importance in mediating this phosphorylation event. In the subsequent investigation of VIK's influence on LBD18 protein stability, cell‐free assays were conducted utilizing GST‐LBD18 recombinant protein. The results showed that the GST‐LBD18 was degraded much faster in the *vik‐2* mutant seedlings extracts compared to the WT counterparts (Figure 7E). We next investigated LBD18 protein stability in vivo using *vik‐2 35S::Myc‐LBD18*, which was generated by crossing *vik‐2* mutant with *35S::Myc‐LBD18* transgenic lines.^[^ ^56^ ^]^ Similar to above cell‐free assays results, LBD18 exhibits a faster degradation rate in *vik‐2* than WT background (Figure 7F). Moreover, we examined LR phenotypes in both *35S::Myc‐LBD18* and *vik‐2 35S::Myc‐LBD18* lines. Interestingly, we observed a substantial attenuation in the LR density increase induced by LBD18 overexpression in the *vik‐2* mutant background (Figure 7G,H), suggesting an interplay between VIK and LBD18 in regulating LR development.

Collectively, these results indicated that VIK directly phosphorylates and stabilizes LBD18 to positively regulate LR development.

## 3 Discussion

Auxin, as one of the most crucial plant hormones, plays a pivotal role in regulating numerous growth and developmental processes in plants.^[^ ^57^, ^58^ ^]^ The perception of auxin and the subsequent signaling transduction are essential prerequisites for realizing its biological functions. The conventional auxin signaling pathway operates via transcriptional regulatory modules mediated by TIR1/AFBs in the nucleus, which involves the TIR1/AFBs‐mediated ubiquitin degradation of AUX/IAAs repressor proteins and releasing the inhibition on ARFs transcription factors.^[^ ^58^, ^59^, ^60^ ^]^ Protein phosphorylation modification‐mediated auxin signaling transduction, directly perceived by Auxin‐Binding Protein 1 (ABP1)/ABP1‐like proteins (ABLs)‐TMK1 signaling modules,^[^ ^43^, ^44^ ^]^ also plays a critical role in many processes,^[^ ^60^, ^61^, ^62^ ^]^ especially the fast responses to auxin.^[^ ^37^, ^38^, ^43^, ^44^, ^45^ ^]^ Although several phosphoproteomics analyses have shown that auxin can trigger substantial protein phosphorylation in plants,^[^ ^38^, ^39^, ^43^, ^44^, ^45^ ^]^ the significant role of post‐translational phosphorylation modifications of proteins in auxin signaling response and mediated growth and development processes is far from fully understood. In this study, we identified C1 clade Raf‐like MAPKKK VIK through protein phosphoproteomics, and proved that auxin‐induced VIK phosphorylation positively regulates LR development. This discovery further substantiates the significant role of protein phosphorylation modifications in auxin signal transduction and its regulation in biological processes.

**Figure 8:**
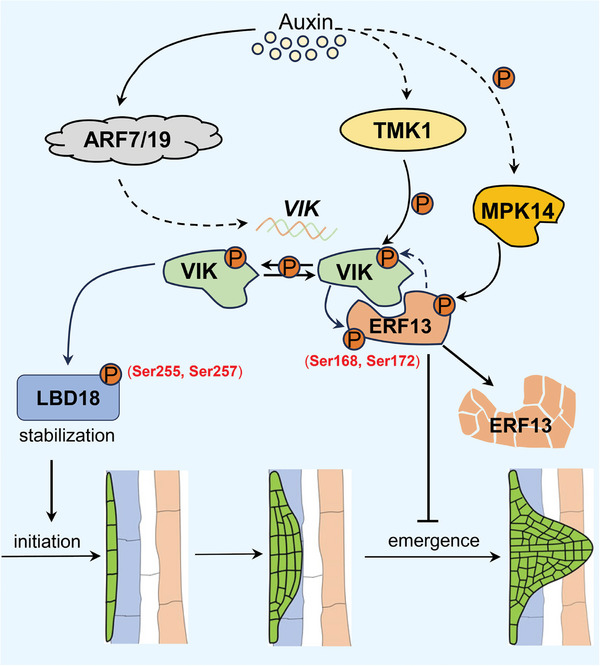
A proposed model illustrating function of VIK‐mediated regulatory modules in LR development. VIK‐mediated auxin signaling regulates LR formation. On one hand, auxin induces the transcription of *VIK* in an ARF7/19‐dependent manner, and on the other hand, it activates the kinase activity of VIK through TMK1. Activated VIK can phosphorylate both LBD18 and ERF13. Phosphorylated LBD18 becomes more stable, regulating the initiation of LR. However, the phosphorylated ERF13 undergoes degradation, relieving its inhibition on LR emergence and consequently promoting LR formation.

In recent years, the significant role of auxin‐triggered protein phosphorylation modifications in LR development has gradually become evident. During LR initiation, auxin signaling activated‐MPK6 appropriately confines asymmetric cell divisions to ensuring LR organogenesis.^[^ ^63^ ^]^ From stage I, LRP cell division patterns are regulated by TMK1/4‐MKK4/5‐MPK3/6 cascade pathway, which acts in downstream of auxin.^[^ ^36^ ^]^ Moreover, MKK4/5‐MPK3/6 module also plays key roles to facilitate LR emergence through upregulating cell wall related genes from stage IV onward.^[^ ^64^ ^]^ In addition, another MPK member MPK14 is activated by auxin signaling to phosphorylate ERF13, thus enhancing its interaction with E3 ligase MAC3A/3B.^[^ ^46^ ^]^ The ubiquitination of ERF13 results in its degradation, thereby relieving the inhibition on LR development.^[^ ^40^ ^]^ Conversely, under drought stress conditions, MPK3/6 directly phosphorylate and stabilizes Aux/IAA15, inhibiting the occurrence of LRs.^[^ ^65^ ^]^ In this study, we present a regulatory model outlining how VIK‐mediated auxin signaling regulates LR development, as illustrated in **Figure** **8**. On one hand, auxin induces the transcription of *VIK* in an ARF7/19‐dependent manner, and on the other hand, it can activate the kinase activity of VIK through TMK1. Activated VIK can phosphorylate both LBD18 and ERF13. Phosphorylated LBD18 becomes more stable, regulating the LR initiation, while phosphorylated ERF13 undergoes degradation, relieving its inhibition on LR emergence and consequently promoting LR formation. Our studies not only elucidate the molecular mechanism by which VIK mediated auxin signaling in LR formation, but also, in conjunction with previous studies, further substantiate the pivotal role of kinase‐mediated auxin signaling in LR development.

Previous investigations have proposed that several Raf‐like MAPKKKs regulate plant growth and development through directly phosphorylating their substrates,^[^ ^66^, ^67^, ^68^, ^69^ ^]^ rather than relying on the stereotypical MAPK cascade pathway. For instance, in the absent of ethylene, Raf‐like MAPKKK CTR1 phosphorylates ETHYLENE‐INSENSITIVE2 (EIN2), a core regulator of ethylene responses. This phosphorylation prevents EIN2 from entering the nucleus to activate ethylene responsive genes.^[^ ^68^ ^]^ In addition, another Raf‐like MAPKKK Raf10 was shown to interact with ABA signaling components such as PP2Cs, SnRK2s, ABI5/ABFs, and ABI3 and regulate ABA signaling.^[^ ^69^ ^]^ Here, we identified C1 clade Raf‐like MAPKKK VIK mediates auxin signaling to regulate LR development through interacting and phosphorylating LBD8 and ERF13, respectively. All these observations suggest that the capability to directly phosphorylate substrates might be an exclusive characteristic of Raf‐like MAPKKKs compared to other MAPKKK members, possibly due to their physical interaction with substrate proteins. Furthermore, substrate phosphorylation independent of the stereotypical MAPK cascade signaling pathway may mediate a rapid or more efficient signal transduction process, ensuring the timely perception and response of downstream components.

B4 clade Raf‐like protein kinases have recently been demonstrated as central mediators of auxin‐triggered phosphorylation across species, highlighting an ancient system for rapid responses to auxin signaling.^[^ ^45^ ^]^ In this study, we discovered that VIK, a C1 clade Raf‐like MAPKKK, is rapidly induced by auxin and plays a crucial role in controlling LR development. This finding further supports the notion that Raf‐like kinases are important mediators of auxin signaling in plants. Notably, a recent study has revealed that ILK5, a close homolog of VIK, demonstrates a comparable expression pattern in roots and displays a similar nucleocytoplasmic localization to VIK.^[^ ^47^ ^]^ Furthermore, the other ILKs also show expression in various root tissues, including xylem, cortex, epidermal layer, and LR.^[^ ^48^ ^]^ Therefore, it becomes intriguing to explore whether VIK and its homologs operate redundantly in the context of LR development.

We demonstrated that VIK phosphorylates ERF13 at Ser168 and Ser172 sites (Figure 4C), whereas Lv et al. recently showed that MPK14 phosphorylated ERF13 at Thr66, Ser67, and Thr124 sites.^[^ ^40^ ^]^ Furthermore, we have also demonstrated that VIK and MPK14 promote the degradation of ERF13 in an additive manner (Figure S13B, Supporting Information), indicating a cooperative regulatory role for VIK and MPK14 in facilitating LR emergence. Furthermore, our results reveal a feedback loop: the phosphorylation of ERF13 by VIK enhances VIK phosphorylation (Figure 4C; Figure S9, Supporting Information), establishing a positive regulatory circuit. The VIK‐LBD18 module also probably exhibits a similar regulatory mechanism (Figure 7C,D), underscoring potent regulatory modules during LR development. We have also delved into the molecular mechanism through which ERF13 enhances VIK kinase activity. Considering VIK's autophosphorylation ability, likely dependent on its homodimerization (Figure S10A–C, Supporting Information), we assumed that ERF13 enhanced VIK kinase activity by increasing the number of VIK homodimers in a substrate‐phosphorylated manner. However, our findings indicated that the formation ability of VIK homodimers does not significantly change when co‐existing with different phosphorylated forms of ERF13 (Figure S10D, Supporting Information). Given the interaction between kinase and substrate can trigger kinase spatial conformational changes, enhancing its catalytic cleavage accessibility and ultimately boosting kinase activity in animals and humans.^[^ ^70^, ^71^, ^72^ ^]^ Therefore, whether this regulatory mechanism is applicable to the VIK‐ERF13/LBD18 regulatory module is a topic worthy of future research.

## 4 Experimental Section

### Plant Materials, Growth, and Treatment

All *Arabidopsis* plants in this study are Columbia (Col‐0) ecotype background. The plants were grown on 1/2 Murashige and Skoog (1/2 MS) medium with 0.8% agar (w/v) at 22 °C under light/dark (16/8) photoperiod. A 10 µM NAA (N0640, sigma) solution was employed for treating the seedlings, with the exception of Figure 2E,F, where a 25 nM NAA solution was used. The working concentrations of CHX (94 271, Amresco) and MG132 (2585, APExBIO) were both set at 200 µM.

### Vector Construction

To generate *ProVIK:VIK‐GFP* vector, the 2805‐bp upstream fragment of the start codon of *VIK* was amplified and cloned into pENTR‐D entry vector (Invitrogen). Subsequently, the coding sequence of VIK‐GFP fusion protein was inserted into the 3′ end of *VIK* promoter. Next, the cloned 1257‐bp downstream fragment of *VIK* stop codon was arranged after GFP coding sequence. For *ProERF13:ERF13‐YFP* vector, the 3274‐bp *ERF13* genomic fragment, including a 2596‐bp promoter fragment upstream of the start codon and the coding sequence of ERF13, was amplified and inserted into pENTR‐D vector. Next, the coding sequence of YFP was inserted into the 3′ end of ERF13 coding sequence. To generate *ProLBD18:LBD18‐YFP* vector, the 2822‐bp upstream fragment of the start codon of *LBD18* was cloned into pENTR‐D vector, followed by the insertion of the coding sequence for LBD18‐GFP fusion protein into the 3′ end of the promoter. Finally, all the resulting fragments were transferred into the destination BGW vector using LR recombinase (Invitrogen).

To generate the *35S::YFP‐VIK* construct, the VIK coding sequence within the pENTRY vector was transferred into the pEarleyGate‐104 destination vector using LR recombinase (Invitrogen). The other overexpression vectors are constructed based on the pGreen II skeleton. For the *35S::ERF13^WT^‐Myc*, *35S::ERF13^2A^‐Myc*, and *35S::ERF13^2D^‐Myc* vectors, the coding sequences of ERF13, ERF13^2A^, and ERF13^2D^ were individually incorporated into the multiple cloning sites, each followed by a 3 × Myc tag. Similarly, for the *35S::ERF13‐GFP*, *35S::ERF13^2A^‐GFP*, and *35S::ERF13^2D^‐GFP* vectors, the coding sequences of ERF13, ERF13^2A^, and ERF13^2D^ were individually inserted into the multiple cloning sites, each followed by a GFP tag. All primers for vector construction were listed in Table S2 (Supporting Information).

### Phosphoproteome Analysis

Ten‐day‐old *Arabidopsis* wild‐type seedlings grown on 1/2 MS medium were treated with 10 µM NAA for 15 min and 8 h. The roots of the treated seedlings were ground into power in liquid nitrogen and total proteins were extracted according to a previous method.^[^ ^73^ ^]^ Subsequent protein digestion, phosphopeptides enrichment, and identification, as well as analysis were performed by Shanghai Center for Plant Stress Biology (Chinese Academy of Sciences) and Shanghai Bioprofile Biotechnology.

### RNA Extraction and qRT‐PCR Analysis

Total RNA was extracted using the RNAsimple Total RNA Kit (DP419, TIANGEN) according to the manufacturer's instructions. The genomic DNA was digested using 4 × gDNA wiper Mix (Vazyme), and HiScript II Select qRT SuperMix II (Vazyme) was applied to synthesize cDNA. Quantitative real‐time PCR (qRT‐PCR) was performed using ChamQ SYBR Color qPCR Master Mix (Vazyme) on a MyiQ Real‐time PCR Detection System (Bio‐Rad, USA). All qRT‐PCR analyses were conducted in triplicate, with *ACTIN2* as the internal reference gene. Primers used in qRT‐PCR analysis are listed in Table S2 (Supporting Information).

### Light Microscopy, GUS Staining, and Confocal Imaging

To visualize LR, 10‐day‐old seedlings underwent treatment following a previously described procedure.^[^ ^56^ ^]^ Observation and imaging were conducted using an Olympus BX53 microscope equipped with a DP72 camera and cellSens Standard 1.6 software. β‐glucuronidase (GUS) staining experiments were carried out in accordance with a previously established protocol.^[^ ^40^ ^]^

For PI staining, 10‐day‐old *Arabidopsis* seedlings were incubated with 1 mg mL^−1^ PI solution (Sangon Biotech) for 5 min. Subsequently, the treated seedlings were placed on microscope slide for observation and imaging using Zeiss LSM880 confocal microscope. During imaging, GFP was excited at a wavelength of 488 nm and detected within a range of 493–594 nm. YFP was excited at 514 nm wavelength and detected within a range of 519–620 nm. For PI staining observation, PI was excited at 488 nm and detected within a range of 600–630 nm.

### Yeast Two‐Hybrid Assay

The pGADT7 (Clontech) or pGBKT7 (Clontech) were linearized using Nde I and EcoR I restriction enzymes. Subsequently, the full‐length sequences, including VIK, MPK14, and coding sequence of TMK1 kinase domain (the 1308‐bp upstream fragment of the termination codon of TMK1), were individually cloned into the multiple cloning sites of pGADT7 vector. Additionally, the full‐length sequences of VIK and ERF13 were separately inserted into pGBKT7 vector. The built‐up vectors were then transformed into AH109 Chemically Competent Cells (WEIDI Biotech), and yeast cells were grown on SD/‐Leu/‐Trp and SD/‐Leu/‐Trp/‐His or SD/‐Leu/‐Trp/‐His/‐Ade medium. The autoactivation was inhibited by the addition of 3‐amino‐1,2,4‐triazole (3‐AT).

### Luciferase Complementation Imaging Assay

For LCI assays, the full‐length sequences of VIK and TMK1 were separately cloned into JW771 vector digested by Kpn I and Sal I restriction enzymes, which generate VIK‐nLUC and TMK1‐nLUC fusion proteins. The coding sequences of VIK and ERF13 were cloned into JW772 respectively, creating cLUC‐VIK and cLUC‐ERF13 fusion proteins. The coding sequences of ERF13, ERF13^2A^, and ERF13^2D^ were then separately inserted into pGreen II vector. To assess VIK‐ERF13 interaction and VIK‐VIK interaction, the indicated combination of GV3101 Agrobacterium cells were co‐transformed into the leaves of *N. benthamiana*. To observe the effect of phosphorylated ERF13 on VIK‐VIK interaction, an equal number of Agrobacterium cells carrying *35S::ERF13* vectors were incorporated into VIK‐VIK combination respectively. The infected tobacco was cultivated in darkness for 24 h, then exposed to light for 48 h. The infected leaves were treated with D‐luciferin potassium salt solution (LUCK‐1G, Gold Biotech) for 10 min, and the LUC activity was visualized using Tanon‐5200 Multi imaging system (Tanon, China).

### Bimolecular Fluorescence Complementation Assay

In BiFC assay, VIK coding sequence was cloned into pSAT6‐nEYFP‐C1 vector creating nYFP‐VIK fusion protein. ERF13 and LBD18 coding sequences were cloned into pSAT6‐cEYFP‐C1 vector generating cYFP‐ERF13 and cYFP‐LBD18 fusion proteins respectively. For VIK‐ERF13 interaction, indicated combination vectors were co‐transformed into *Arabidopsis* mesophyll protoplasts. The following experimental operation were performed as described previously.^[^ ^74^ ^]^ For VIK‐LBD18 interaction, targeted proteins were co‐expressed in leaf cells of *N. benthamiana*. The subsequent procedures were detailed in the preceding LCI assay section. All observation and imaging were operated using Zeiss LSM880 confocal microscope.

### Co‐Immunoprecipitation Assay

The *35S::ERF13‐Myc* vector was constructed in a previous study and above‐mentioned *35S::YFP‐VIK* vector was used in co‐IP assay,^[^ ^40^ ^]^ and the procedures as described in a previous study.^[^ ^41^ ^]^ ERF13‐Myc and YFP‐VIK were co‐expressed in *Arabidopsis* mesophyll protoplasts, with ERF13‐Myc or YFP‐VIK being individually expressed as negative controls. Total protein extraction was carried out using NP‐40 lysis buffer (Beyotime Biotech) containing 1 mm phenylmethylsulfonyl fluoride (PMSF). Then the extracts were incubated with Myc‐Trap agarose beads (ytma‐20, ChromoTek) at 4 °C for 2 h. After washing three times with dilution buffer (10 mm Tris‐HCl, pH 7.5, 150 mm NaCl, and 0.5 mm EDTA), enriched proteins on beads were detected by western blot. YFP‐tag and Myc‐tag were recognized by anti‐GFP antibody (HT801‐02, TransGen Biotech) and anti‐Myc antibody (AE010, ABclonal) respectively.

### In Vitro *Pull‐Down*

The coding sequences of VIK and LBD18 were cloned into pET‐30a and pGEX‐4T‐1 vectors to generate recombinant His‐VIK and GST‐LBD18, respectively. These recombinant proteins were expressed in *Escherichia coli* BL21 cells (DE3), and purified using Ni NTA Beads (Smart‐Lifesciences Biotech) and Glutathione Beads (Smart‐Lifesciences Biotech) according to corresponding to their respective manuals. GST‐LBD18 was immobilized on Glutathione Beads and then incubated with His‐VIK or His‐MBP at 4 °C for 4 h. Subsequently, the Glutathione Beads were washed five times with 1 × PBS buffer. The proteins bound to the beads were analyzed by western blot. His‐tag and GST‐tag were detected by anti‐His antibody (HT501, TransGen Biotech) and anti‐GST antibody (HT601, TransGen Biotech) respectively.

### In Vitro Phosphorylation Assay

The coding sequence of ERF13 was cloned into pGEX‐4T‐1 vector to generate GST‐ERF13 fusion protein. Similarly, recombinant GST‐ERF13^S168A^, GST‐ERF13^S168D^, GST‐ERF13^S172A^, GST‐ERF13^2A^, GST‐ERF13^2D^, GST‐ERF13^3D^, GST‐ERF13^5D^ and GST‐LBD18^S255/257A^ were obtained as GST‐ERF13. The coding sequence of TMK1^KD^ was cloned into pSuper1300‐GFP vector to produce TMK1^KD^‐GFP fusion protein, subsequently expressed in *Arabidopsis* protoplast. The in vitro phosphorylation experiments were operated as described previously.^[^ ^74^ ^]^ Recombinant His‐VIK was used to incubate with GST‐ERF13 or other mutant forms in the reaction buffer [50 mm Tris‐HCl, pH 7.5, 10 mm MgCl_2_, 1 mm DTT, and 1 mm ATP‐gamma‐S (ab138911, abcam)] at 30 °C for 1 h. Then 20 mM EDTA and p‐Nitrobenzyl mesylate (ab138910, abcam) were added to terminate the reaction. The phosphorylated proteins were detected using anti‐thiophosphate ester (TPE) antibody (ab92570, abcam).

For another in vitro phosphorylation analysis using Phos‐tag SDS‐PAGE gel, the experimental procedures were performed as described previously.^[^ ^40^ ^]^ In brief, total proteins were extracted from 10‐day‐old *Arabidopsis* seedlings using non‐denaturating extraction buffer [10 mm NaCl, 25 mm Tris‐HCl, pH 7.5, 1 mm PMSF, 10 mm ATP, 5 mm DTT, and 10 mm MgCl_2_], and then incubated with recombinant His‐VIK for 15, and 30 min respectively. The proteasome inhibitor MG132 and protease inhibitor (04 693 159 001, Roche) were used to inhibit protein degradation. The PhosSTOP reagent (04 906 837 001, Roche) was used to block phosphatase activity. The reaction was terminated with SDS loading buffer. Calf intestinal alkaline phosphatase (CIAP) (M0525S, NEB) was used to inhibit phosphorylation of substrate.

### Phosphorylation Mass Spectrometry Assay

The phosphorylated sites in ERF13 and LBD18, as recognized by VIK, were identified by an in vitro phosphorylation mass spectrometry assay.^[^ ^74^ ^]^ The in vitro reaction system containing His‐VIK, GST‐ERF13/GST‐LBD18 and above‐mentioned reaction buffer was incubated at 30 °C for 1 h. The potential phosphorylation sites were then identified using LC‐MS/MS analysis performed by Shanghai Bioprofile Biotechnology.

### Cell‐Free Protein Degradation Assay

To monitor the degradation of target proteins, cell‐free assays were used and performed according to previous method.^[^ ^74^ ^]^ Total proteins were extracted from 10‐day‐old *Arabidopsis* seedlings with non‐denaturating extraction buffer [10 mm NaCl, 25 mm Tris‐HCl, pH 7.5, 1 mm PMSF, 10 mm ATP, 5 mm DTT, and 10 mm MgCl_2_], and incubated with recombinant proteins for different time periods. The target proteins were detected with corresponding antibody.

### Accession Numbers

All genes sequence information in this article can be found in Arabidopsis Genome Initiative according to accession numbers as follows: *VIK* (AT1G14000), *ERF13* (AT2G44840), *LBD18* (AT2G45420), *LBD16* (AT2G42430), *LBD29* (AT3G58190), *LBD33* (AT5G06080), *ARF7* (AT5G20730), *ARF19* (AT1G19220), *GATA23* (AT5G26930), *EXP14* (AT1G19220), *KCS8* (AT2G15090), *KCS16* (AT2G15090), *E2Fa* (AT2G36010) and *TMK1* (AT1G66150).

### Statistical Analysis

Both Student's t‐test and one‐way ANOVA were performed using SPSS software (version‐21, IBM) to evaluate the significance. For Student's *t*‐test, the *P*‐value < 0.05 indicates a significant difference. *P*‐value < 0.05 is denoted by (*) and *P*‐values < 0.01 is denoted by (**). For one‐way ANOVA, the *P*‐value < 0.05 indicates a significant difference. The details of the statistical analysis of the experiment, including specific testing methods and n‐values, are presented in figure legends.

## Conflict of Interest

The authors declare no conflict of interest.

## Author Contributions

Z.D., H.T., and E.S. planned and designed the experiments. E.S., K.W., B.L., X.Z., X.L., Z.H.D., and J.L. performed the major experiments; Z.D., H.T., and E.S. wrote the paper.

## Supporting information

Supporting Information

Supplemental Table 1

Supplemental Table 2

## Data Availability

The data that support the findings of this study are available in the supplementary material of this article.
