# Supplementary material for: VIK‐Mediated Auxin Signaling Regulates Lateral Root Development in Arabidopsis
Source: Adv Sci (Weinh). 2024 Jul 3;11(33):2402442. doi: 10.1002/advs.202402442 (PMC11434109; doi:10.1002/advs.202402442)
Supplement: Supplementary file 1 — Supporting Information [file ADVS-11-2402442-s001.docx]

Supporting Information

VIK-mediated Auxin Signaling Regulates Lateral Root Development in *Arabidopsis*

Erlei Shang, Kaijing Wei, Bingsheng Lv, Xueli Zhang, Xuefeng Lin, Zhihui Ding, Junchen Leng, Huiyu Tian, Zhaojun Ding*

**
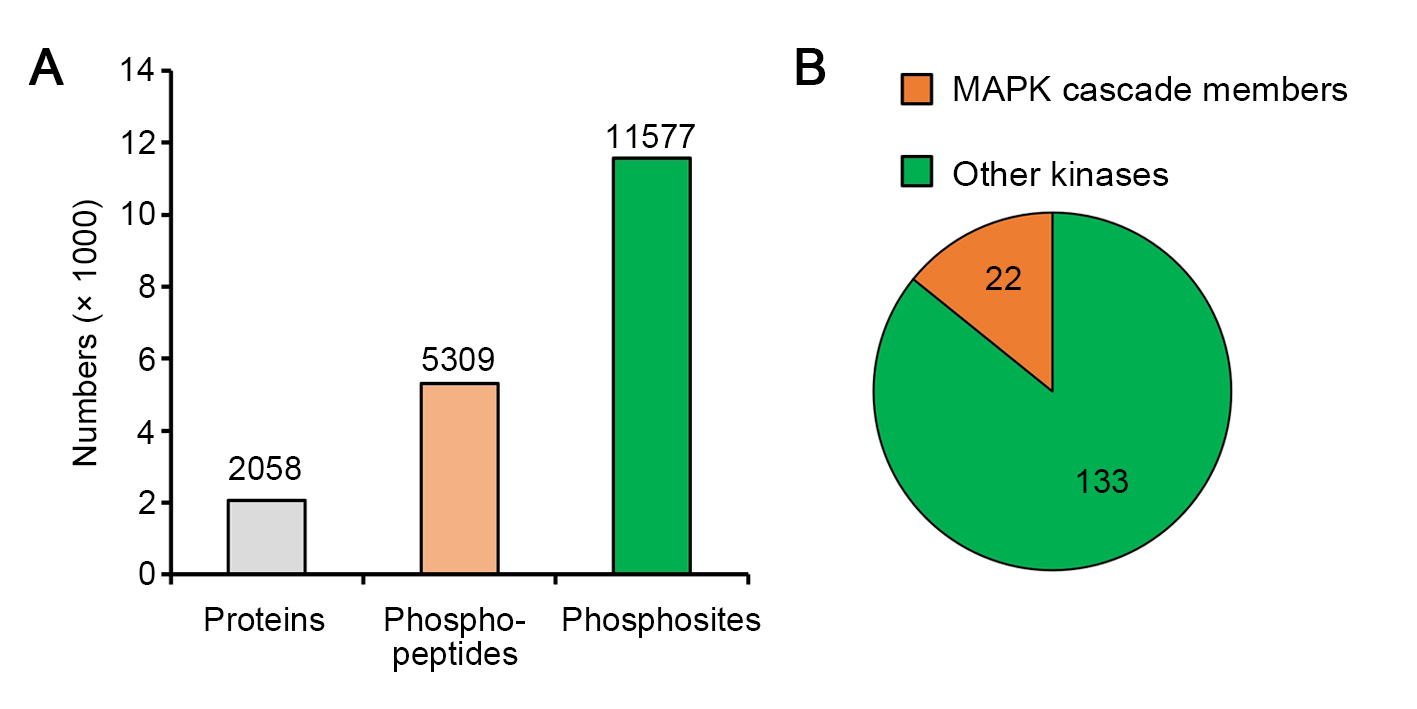
**

**Figure S1**.

Results of phosphoproteomics analysis. A) Numbers of digested phosphopeptides, identified phosphosites and corresponding proteins from root total protein of 10-day-old WT seedlings. B) The number of kinases and the proportion of MAPK cascade members in identified kinases.

**
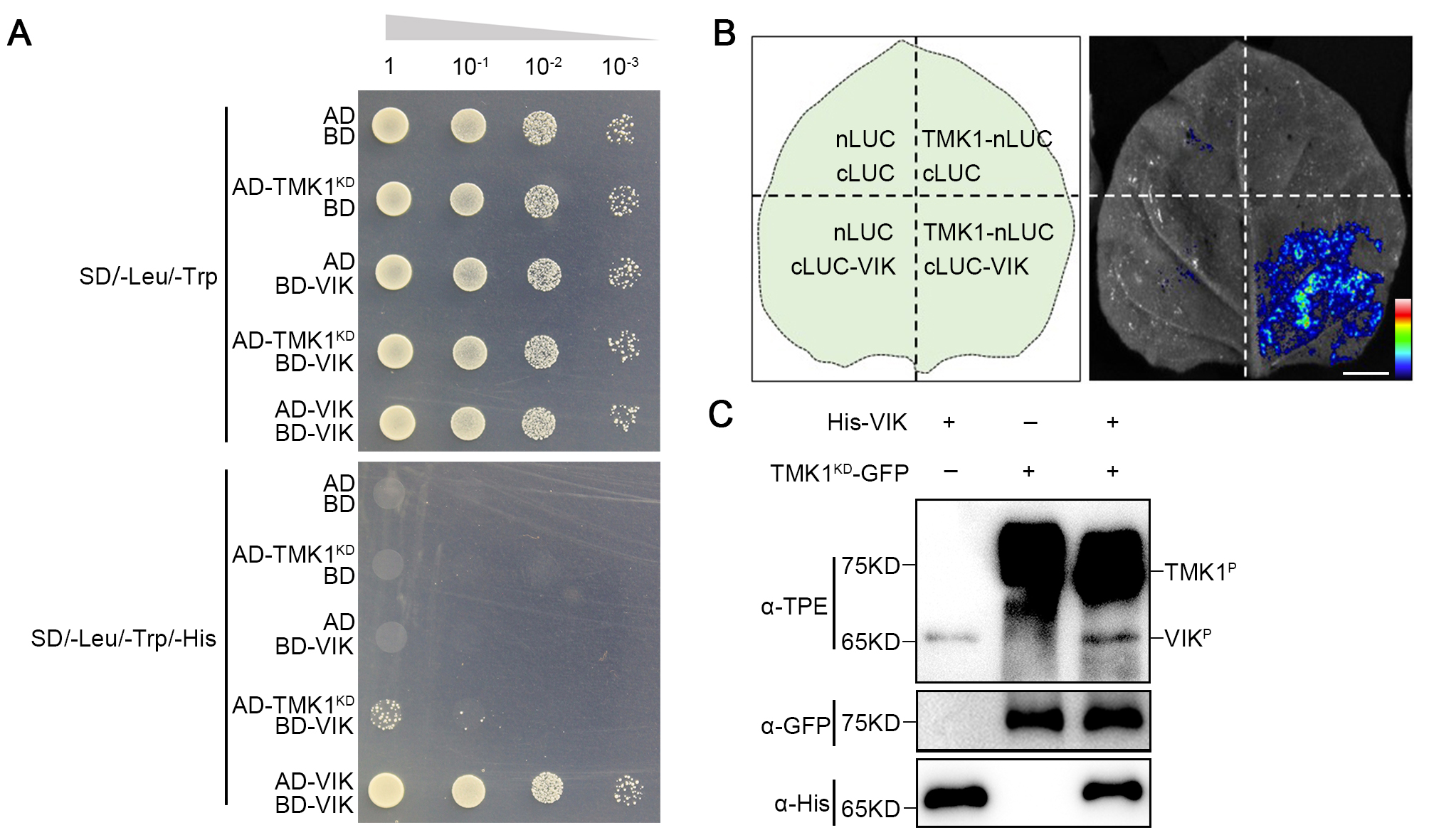
**

**Figure S2**.

TMK1 phosphorylates VIK protein. A) Y2H assay showing interaction between VIK and TMK1. TMK1 kinase domain (TMK1^KD^, 507-942aa) was utilized as the prey in the yeast two-hybrid (Y2H) system by fusing it to the C-terminal of the activation domain (AD). The full-length VIK served as the bait by fusing it to the C-terminal of binding domain (BD). Yeast cells were grown on synthetic dextrose minimal medium without leucine and tryptophan (SD/-Leu/-Trp) and synthetic dextrose minimal medium without leucine, tryptophan and histidine (SD/-Leu/-Trp/-His) supplemented with 1 mM 3-amino-1,2,4-triazole (3-AT). AD or BD represents empty vector. Paired plasmids AD-VIK and BD-VIK was used as positive control. B) Luciferase complementation imaging (LCI) assay. The full-length TMK1 and VIK were fused to nLUC and cLUC to create TMK1-nLUC and cLUC-VIK respectively. The built-up vectors were co-transformed into leaves of *Nicotiana benthamiana*. The color column indicates the range of luminescence intensity. Scale bar, 1 cm. C) The in vitro phosphorylation assay showed that VIK was phosphorylated by TMK1^KD^. His-VIK and TMK1^KD^-GFP recombinant proteins were expressed in *Escherichia coli* and *Arabidopsis* protoplasts, respectively. Phosphorylated proteins were detected using anti-TPE antibody. TMK1^KD^-GFP and His-VIK proteins were detected using anti-GFP and anti-His respectively.


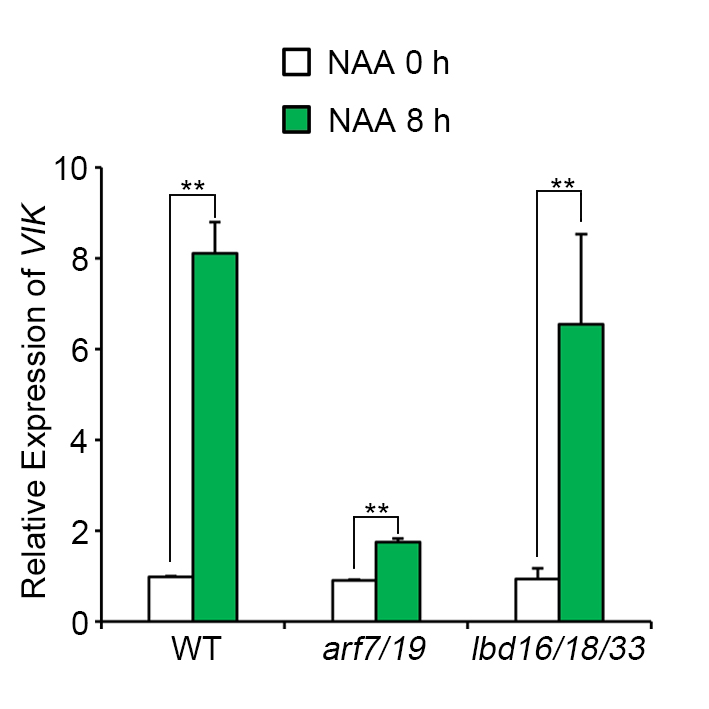


**Figure S3**.

The relative transcription levels of *VIK* in roots. The *VIK* mRNA levels were assessed in roots of 10-day-old WT, *arf7/19* and *lbd16/18/33* mutants. All seedlings were treated with 10 μM NAA for 0 h and 8 h respectively. The expression levels were quantified by quantitative real-time PCR (qRT-PCR) and *ACTIN2* served as an internal control. Error bars indicate SD of three biological replicates. Asterisks represent significant differences compared to WT used Student’s *t* test (***P* < 0.01).


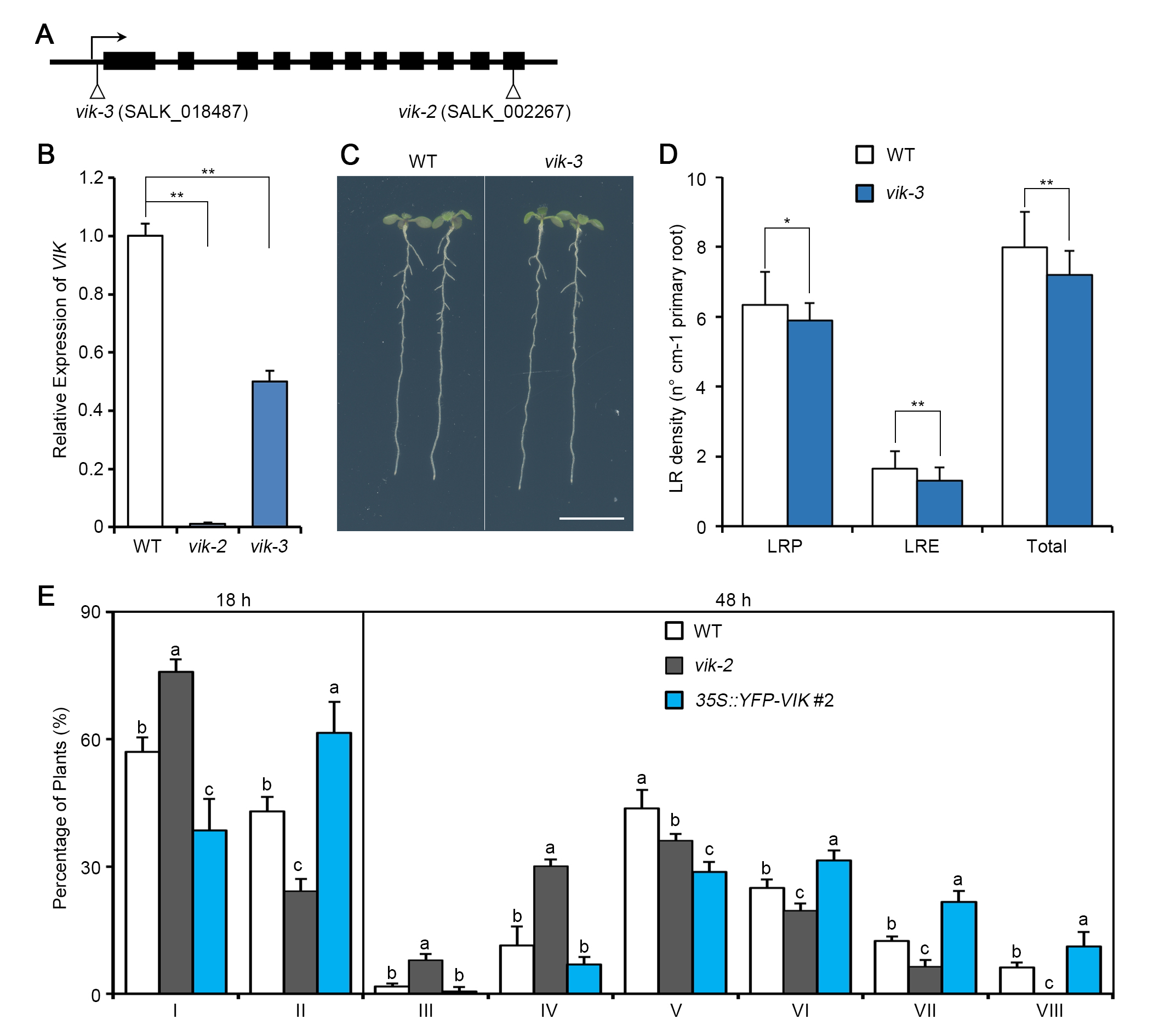


**Figure S4**.

The *vik* mutant showing defects in LR development. A) Schematic diagram illustrating the T-DNA insertion sites of *vik-2* and *vik-3* in *VIK* locus. B) *VIK* mRNA levels in roots of 10-day-old seedlings of two independent mutants *vik-2* and *vik-3*. The expression levels were quantified by qRT-PCR and *ACTIN2* served as an internal control. Error bars indicate SD of three biological replicates. Asterisks represent significant differences compared to WT used Student’s *t* test (***P* < 0.01). C) LR phenotypes of 10-day-old WT and *vik-3* mutant seedlings. Scale bar, 1 cm. D) LR density of 10-day-old WT and *vik-3* mutant seedlings. LR density refers to the ratio of the number of LR to primary root length. LRP, LRE and Total represents LR primordia, emerged LR and total LR, respectively. Data are indicated as means ± SD (*n* =30). Asterisks represent significant differences compared to WT used Student’s *t* test (**P* < 0.05 and ***P* < 0.01). E) Proportion of LRP developmental stages after gravistimulation in 3-day-old WT, *vik-2* and *35S::YFP-VIK* seedlings at 18 and 48 h. Error bars indicate SD of three biological replicates. Different letters indicate significant differences in an independent developmental stage used one-way ANOVA (*P* < 0.05).

**
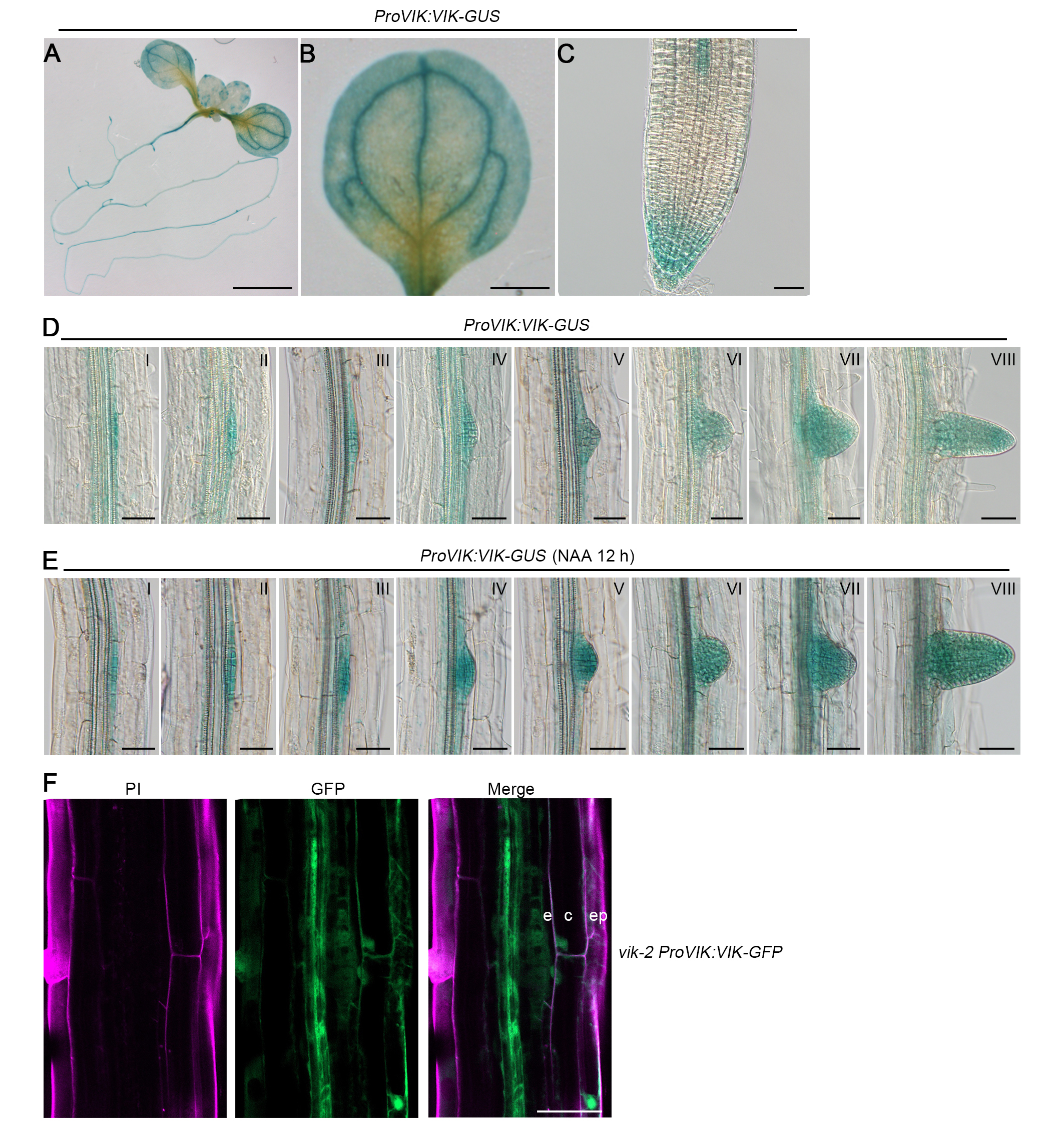
**

**Figure S5.**

The expression pattern of *VIK*. A-E) The expression of VIK in 10-day-old *ProVIK:VIK-GUS* transgenic lines. A-D) VIK is expressed mainly in leaf venation (B), primary root tip (C), stele and all LRs at different stage (D). E) NAA enhances *VIK* expression in all LRs. Ten-day-old seedlings were transferred to 1/2 MS medium (D) and medium supplemented with 10 μM NAA (E) for 12 h, respectively. F) VIK was expressed in LR and endodermis (e), cortex (c) and epidermis (ep) cells of primary root of 10-day-old *vik-2* *ProVIK:VIK-GFP* transgenic lines. Green and purple represent GFP signal and PI staining cell boundary, respectively. Scale bars: 4 mm in (A), 2.5 mm in (B), 50 μm in (C) and 40 μm in (D-F).


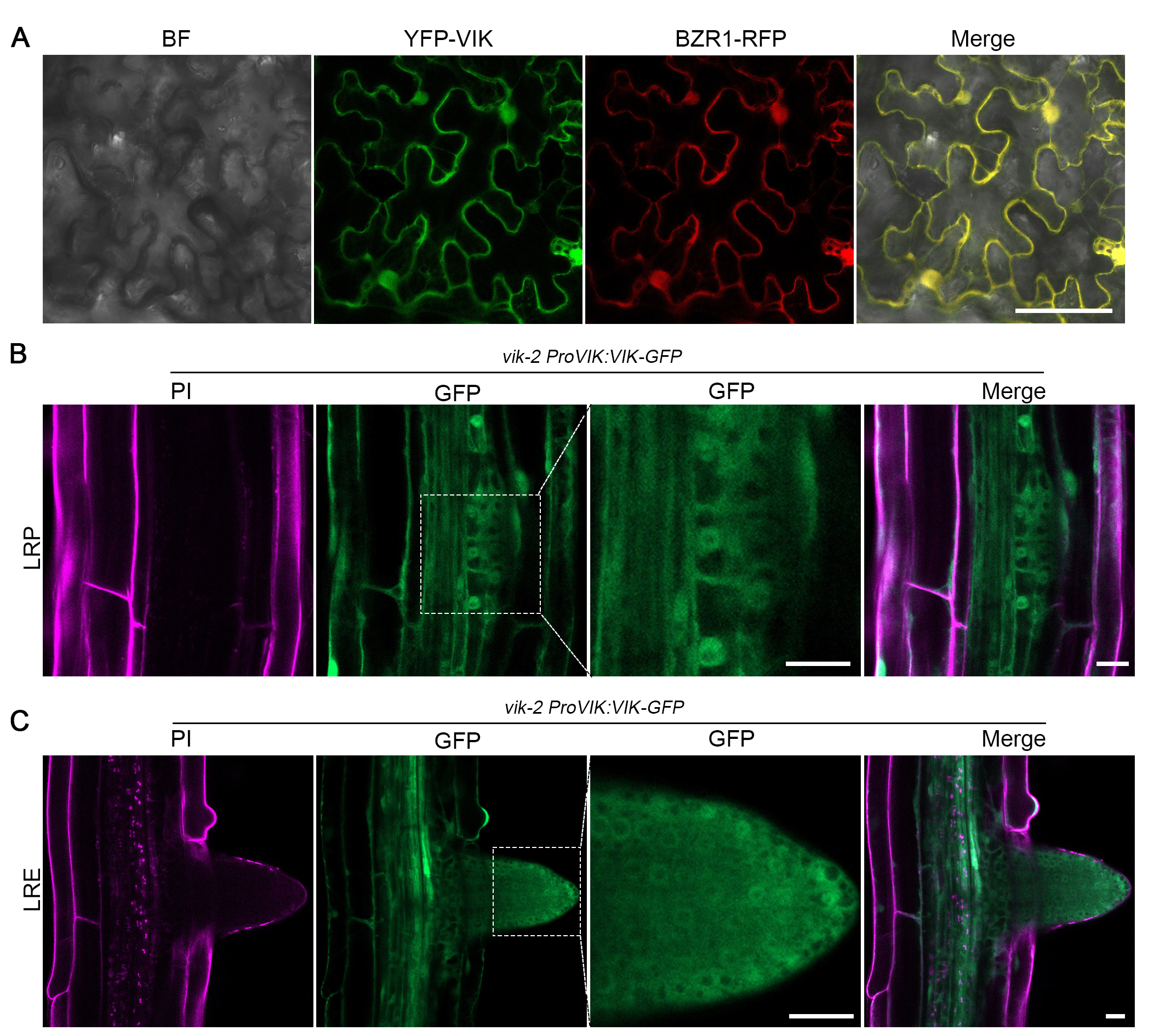


**Figure S6**.

Subcellular localization of VIK protein. A) Confocal images of YFP-VIK and BZR1-RFP transiently co-expressing in leaf cells of *N. benthamiana*. BZR1-RFP served as nucleo-cytoplasmic localization marker. Green and red represent YFP and RFP signals, respectively. BF represents bright field. B and C) VIK-GFP in LRP (B) and LRE cells (C) of 10-day-old *vik-2 ProVIK:VIK-GFP* transgenic plants. The white dotted line boxes indicate the close-up views. Green and purple represent GFP signal the PI staining cell boundary respectively. Scale bars: 50 μm in (A), 20 μm in (B) and (C).


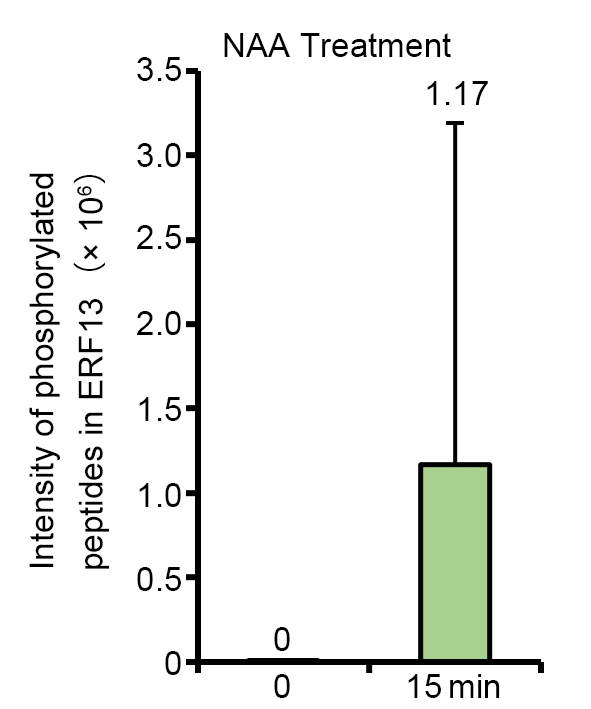


**Figure S7**.

The phosphorylation of ERF13 was triggered by auxin observed in phosphorylomics analysis. The phosphopeptides intensity of ERF13 from root total protein of 10-day-old WT seedlings with or without NAA treatment (10 μM NAA for 15 min).

**
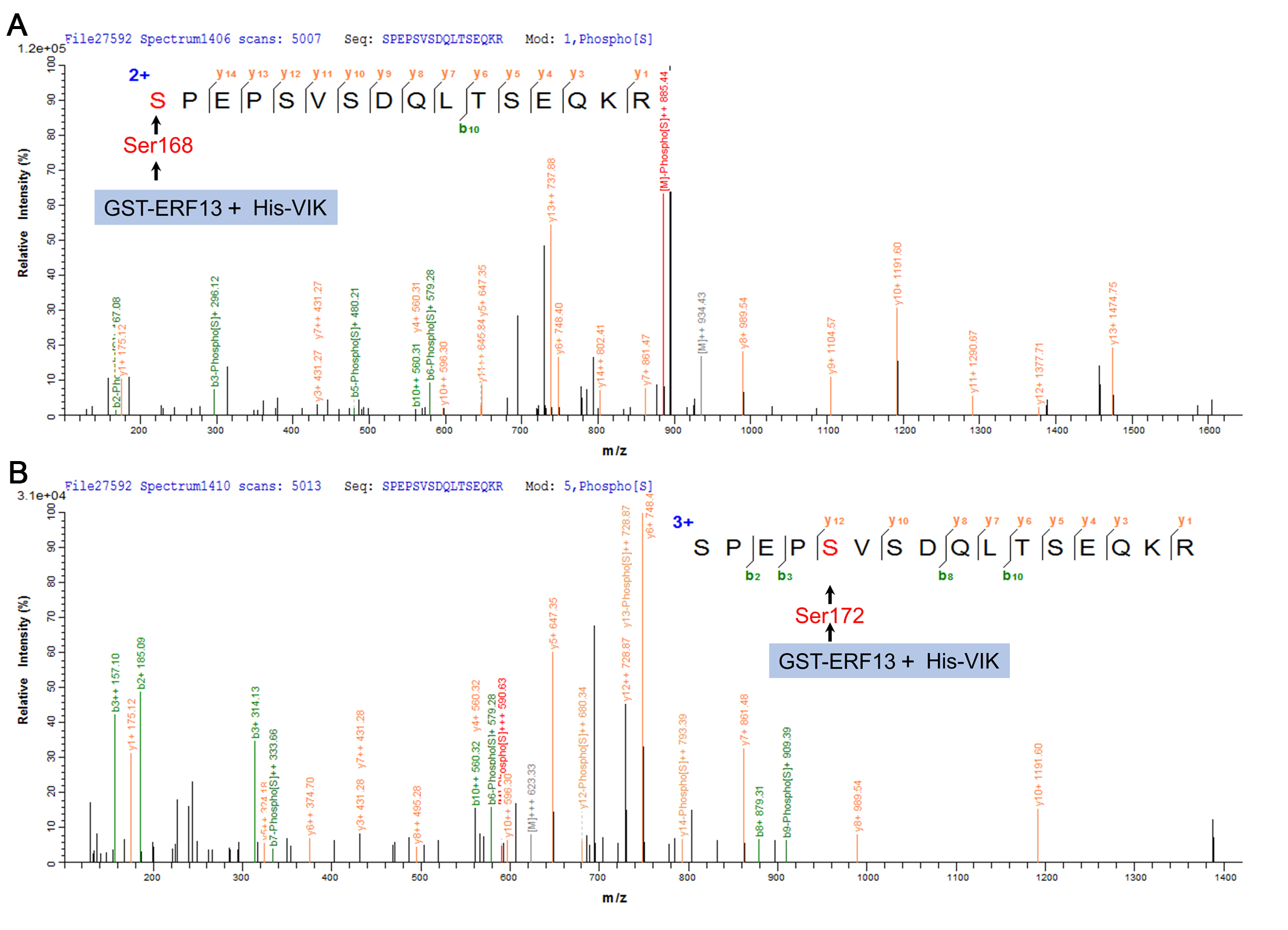
**

**Figure S8**.

The relative abundances of phosphopeptides in ERF13 detected by phosphorylation-mass spectrum assay. Recombinant GST-ERF13 was incubated with His-VIK in reaction buffer containing 10 mM ATP. The peaks marked in red were denoted phosphorylated amino acid residues Ser168 (A) and Ser172 (B) in two peptide fragments of ERF13 protein in phosphorylation-mass spectrum assay. The ordinate and abscissa indicate relative intensity and mass charge ratio (m/z) respectively.

**
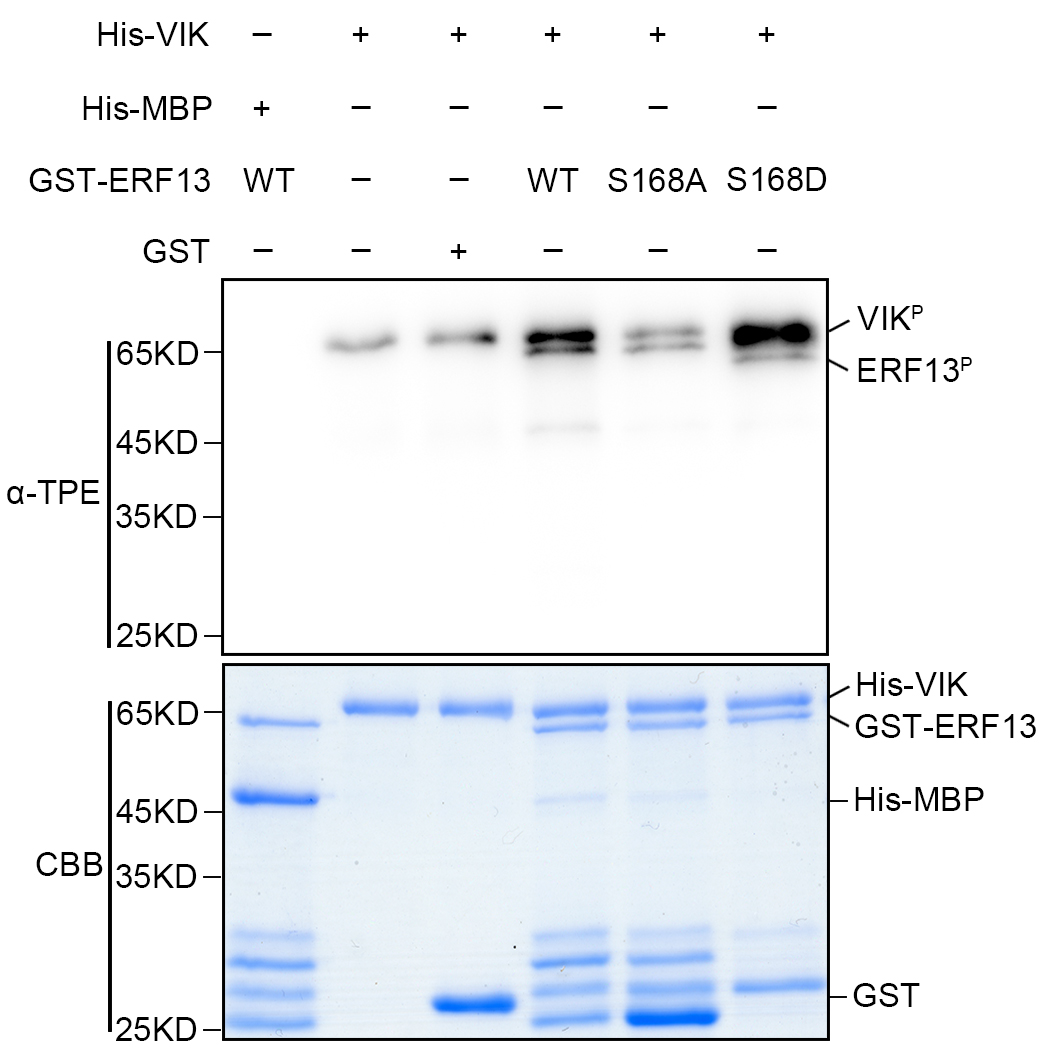
**

**Figure S9.**

The in vitro phosphorylation assay showing that ERF13 enhances VIK autophosphorylation level. In vitro, recombinant GST-ERF13, GST-ERF13^S168A^ and GST-ERF13^S168D^ were separately incubated with His-VIK in a reaction buffer. Phosphorylated proteins were detected using anti-TPE antibody. GST and His-MBP served as negative control. The loading proteins were visualized by Coomassie brilliant blue (CBB) staining.

**
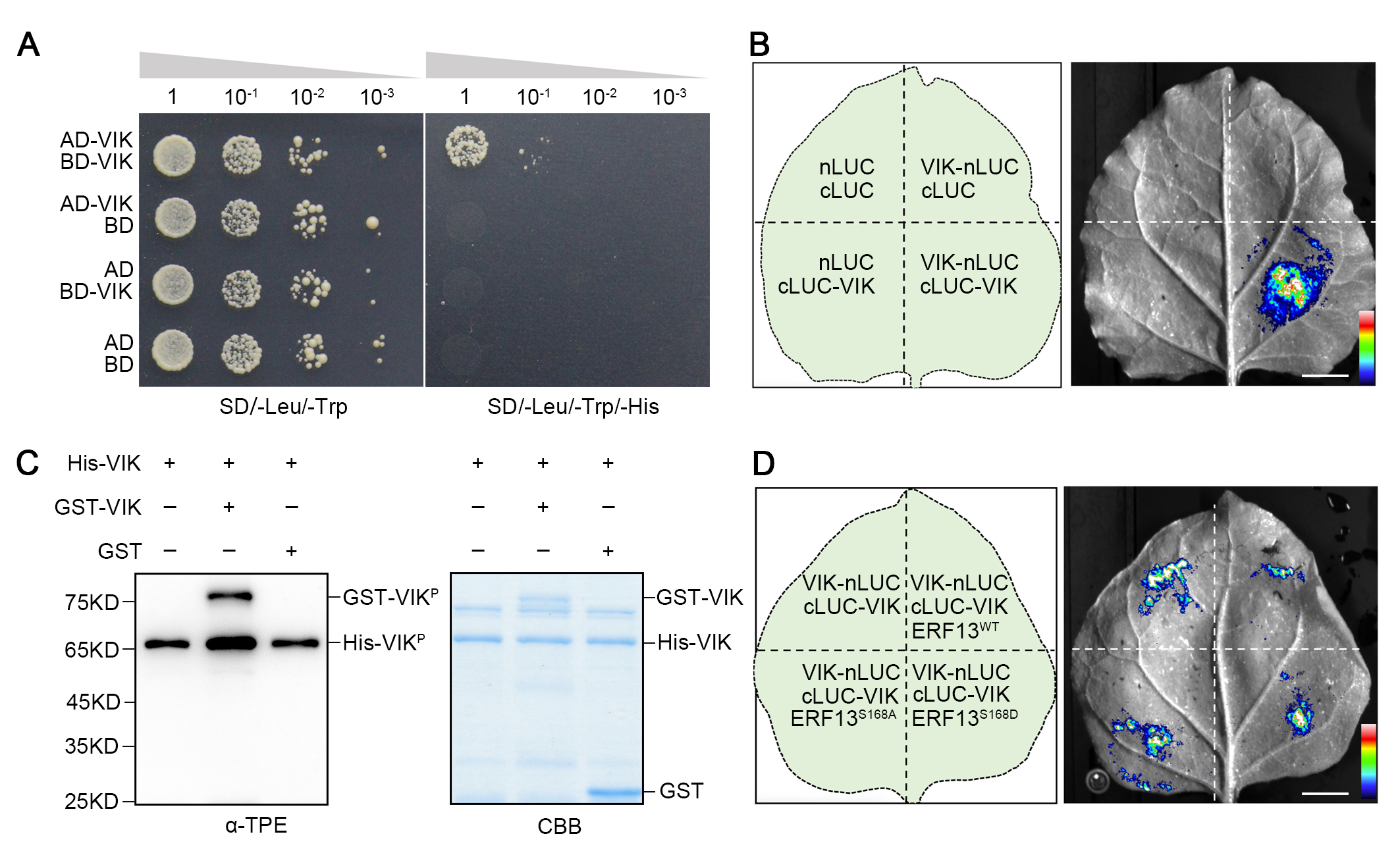
**

**Figure S10.**

ERF13 doesn’t affect the formation of VIK homodimer. A) Y2H assay showing VIK-VIK interaction. Yeast cells were grown on SD/-Leu/-Trp and SD/-Leu/-Trp/-His medium supplemented with 5 mM 3-AT. AD or BD represents empty vector. B) LCI assay. The full-length VIK were fused to nLUC and cLUC to create VIK-nLUC and cLUC-VIK respectively. The built-up vectors were co-transformed into leaves of *N. benthamiana*. C) The in vitro phosphorylation assay showing the ability of VIK autophosphorylation. Phosphorylated proteins were detected using anti-TPE antibody. GST protein served as negative control. The loading proteins were visualized by CBB staining. D) Competitive LCI assays. ERF13, ERF13^S168A^ and ERF13^S168D^ were transiently co-expressed with VIK-nLUC + cLUC-VIK combination in leaves of *N. benthamiana* respectively. The color columns indicate the range of luminescence intensity in (B) and (D). Scale bars, 1 cm in (B) and (D).

**
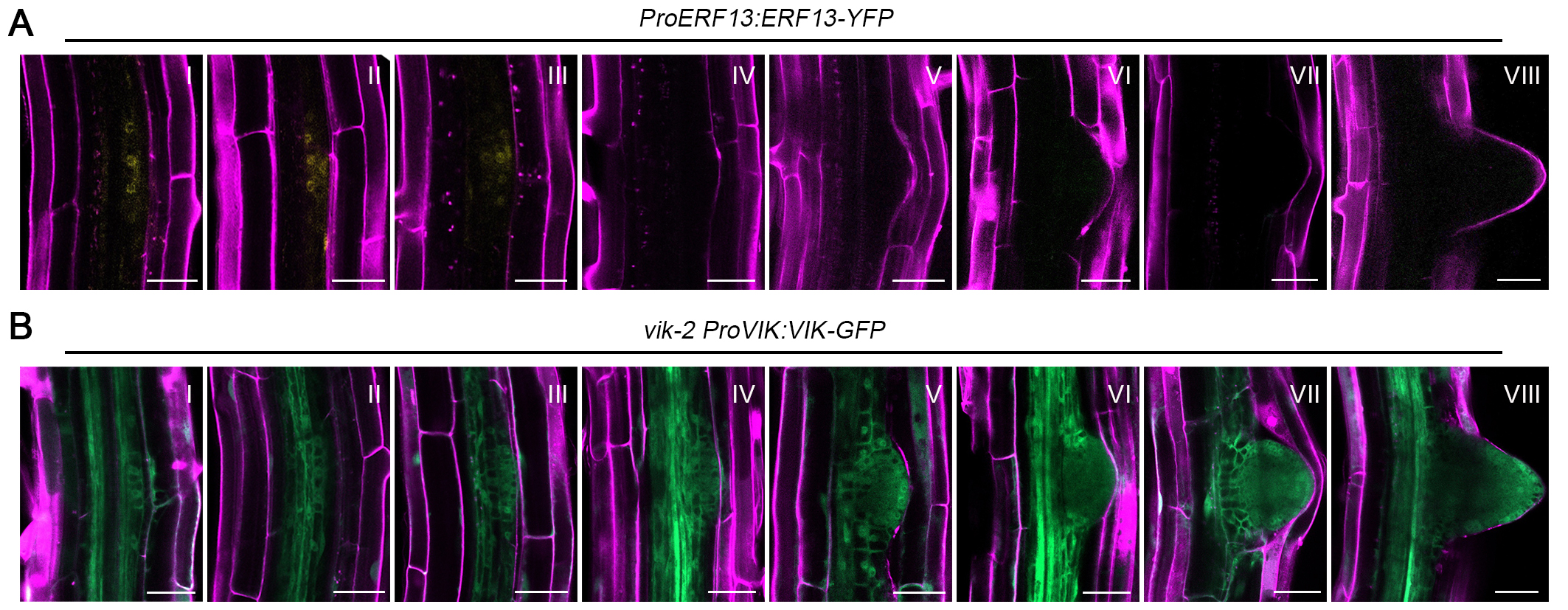
**

**Figure S11**.

The expression patterns of ERF13 and VIK in LRs. A) The expression pattern of ERF13 (yellow) in LRs from 10-day-old *ProERF13:ERF13-YFP* transgenic plants. B) The expression pattern of VIK (green) in LRs from 10-day-old *vik-2* *ProVIK:VIK-GFP* transgenic plants. The roman numeral indicates developmental stage of LR. Yellow and green represent YFP and GFP signals respectively. Purple indicates PI staining cell boundary. Scale bars, 40 μm.

**
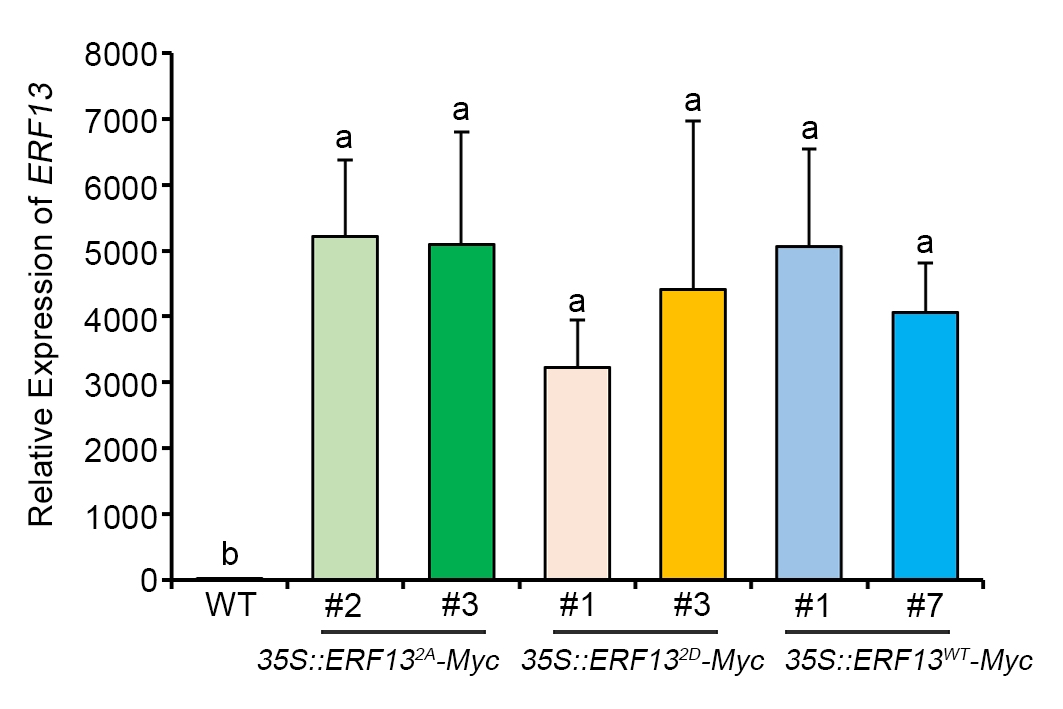
**

**Figure S12**.

Transcript levels of *ERF13* in roots of WT and *ERF13* overexpression lines. The *ERF13* mRNA levels were assessed in roots of 10-day-old WT and *ERF13* overexpression seedlings. ERF13^2A^ and ERF13^2D^ indicate two amino acids sites, Ser168 and Ser172, within ERF13, are substituted with alanine (A) and aspartic acid (D), respectively.The mRNA levels were quantified by qRT-PCR and *ACTIN2* served as an internal control. Error bars indicate SD of three biological replicates. Different letters indicate significant differences used one-way ANOVA (*P* < 0.05).

**
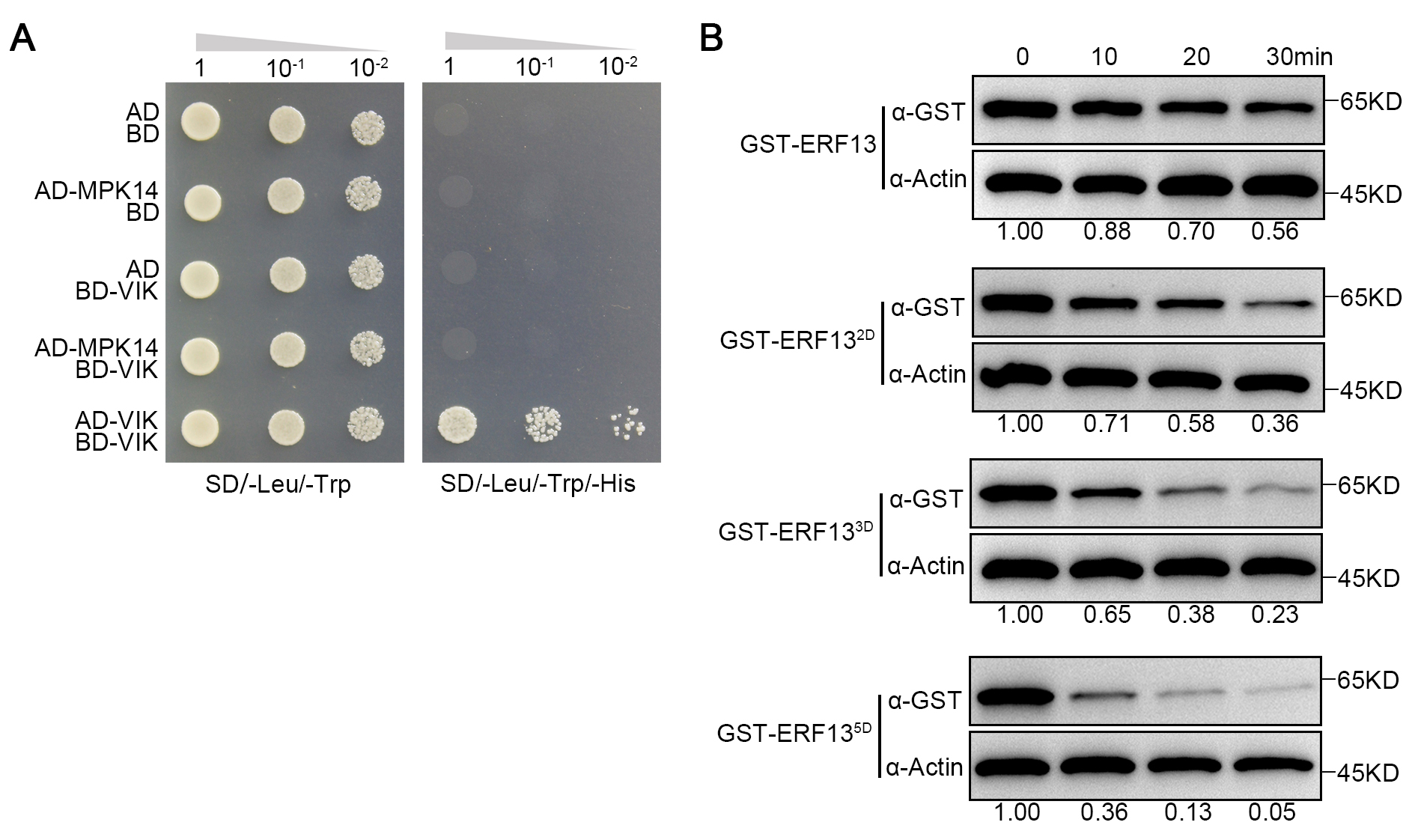
**

**Figure S13**.

VIK and MPK14 cooperatively regulate the degradation of the ERF13 protein. A) Y2H assay showing no interaction between VIK and MPK14. The full-length MPK14 was utilized as the prey in the yeast two-hybrid system by fusing it to the C-terminal of the activation domain (AD). The full-length VIK served as the bait by fusing it to the C-terminal of binding domain (BD). Yeast cells were grown on SD/-Leu/-Trp and SD/-Leu/-Trp/-His medium supplemented with 1 mM 3-AT. AD or BD represents empty vector. Paired plasmids AD-VIK an BD-VIK was used as positive control. B) Cell-free assay showing the phosphorylation of ERF13 mediated by VIK and MPK14 cooperatively promotes the degradation of ERF13. The total proteins were extracted from 10-day-old WT seedlings and incubated with recombinant GST-ERF13, GST-ERF13^2D^ and GST-ERF13^3D^ and GST-ERF13^5D^ for 0, 10, 20 and 30 min, respectively. GST-ERF13 protein was detected by anti-GST antibody. Actin served as a loading control. The abundance of ERF13 relative to Actin was calibrated to 1.00 at 0 h.

**
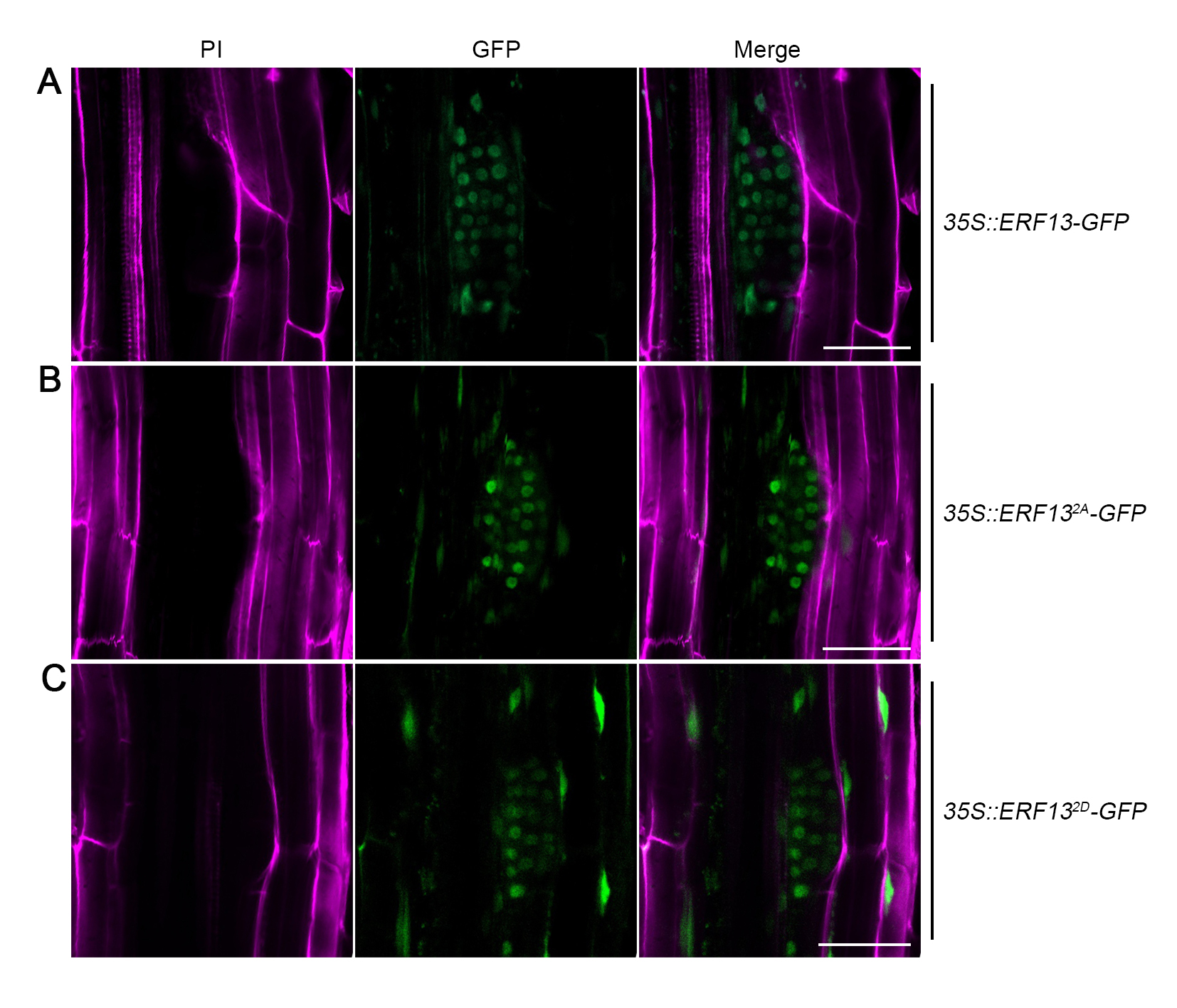
**

**Figure S14**.

ERF13 subcellular distribution pattern was not influenced by VIK-mediated phosphorylation. Confocal images of GFP signals in LRs of 10-day-old *35S::ERF13-GFP* (A), *35S::ERF13^2A^-GFP* (B), and *35S::ERF13^2D^-GFP* seedling roots (C). Green and purple represent GFP signal and PI staining cell boundary, respectively. ERF13^2A^ and ERF13^2D^ indicate two amino acids sites, Ser168 and Ser172, within ERF13, are substituted with alanine (A) and aspartic acid (D), respectively. Scale bars: 40 μm.

**
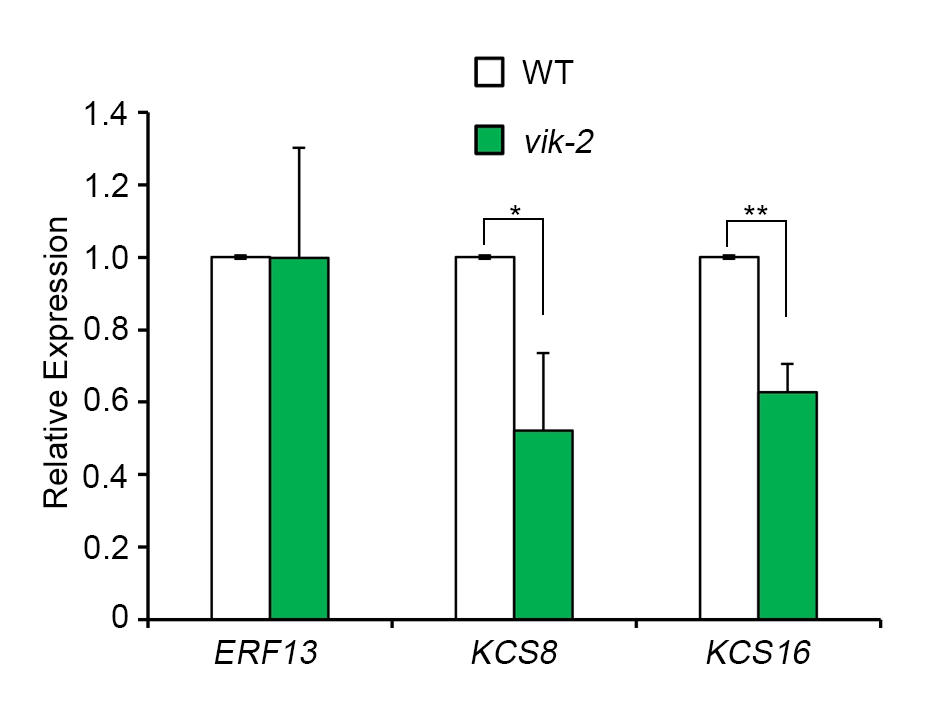
**

**Figure S15**.

Transcript levels of *ERF13*, *KCS8* and *KCS16* in roots of WT and *vik-2* seedlings. The mRNA levels of *ERF13*, *KCS8* and *KCS16* in roots of 10-day-old WT and *vik-2* were quantified by qRT-PCR and *ACTIN2* served as an internal control. Error bars indicate SD of three biological replicates. Asterisks represent significant differences compared to WT used Student’s *t* test (***P* < 0.01 and **P* < 0.05).

**
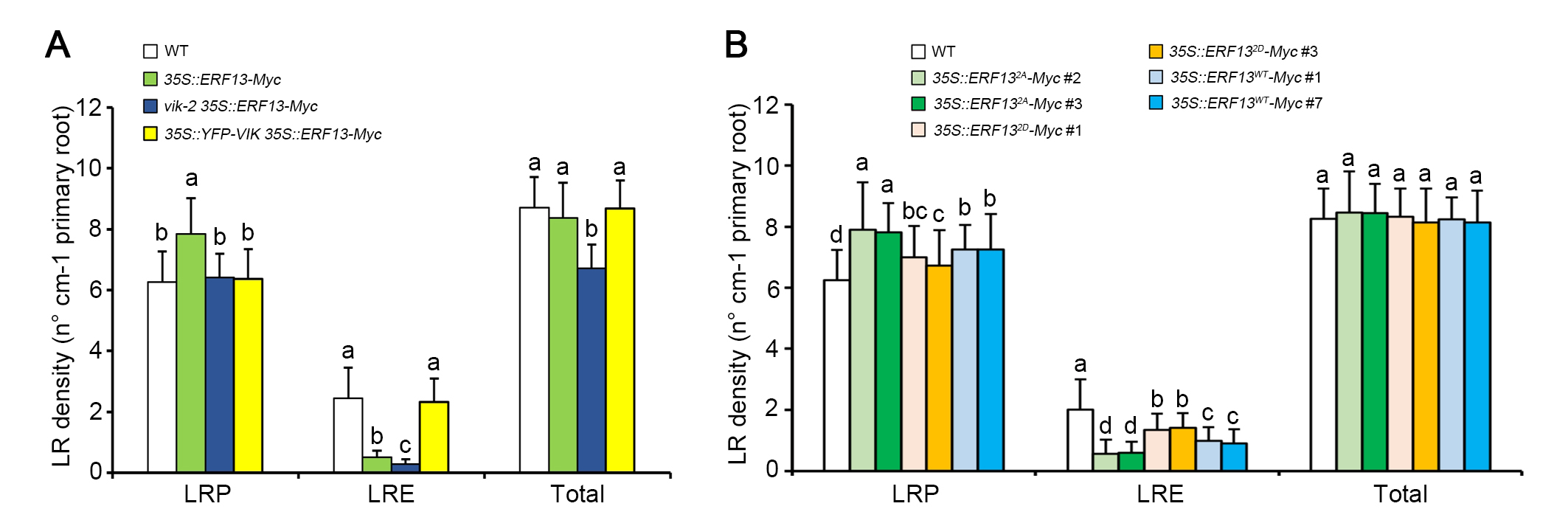
**

**Figure S16**.

VIK-mediated phosphorylation of ERF13 promotes LR emergence. A) LR density in 10-day-old seedlings of WT, *35S::ERF13-Myc*, *vik-2 35S::ERF13-Myc* and *35S::YFP-VIK 35S::ERF13-Myc*. Data are indicated as means ± SD (*n* = 40). B) LR density in 10-day-old seedlings of WT, *35S::ERF13^2A^-Myc*, *35S::ERF13^2D^ -Myc* and *35S::ERF13^WT^-Myc*. LRP, LRE and Total represent LR primordia, emerged LR and total LR, respectively. LR density refers to the ratio of the number of LR to primary root length. Data are indicated as means ± SD (*n* = 40). Different letters indicate significant differences used one-way ANOVA (*P* < 0.05) in both (A) and (B).

**
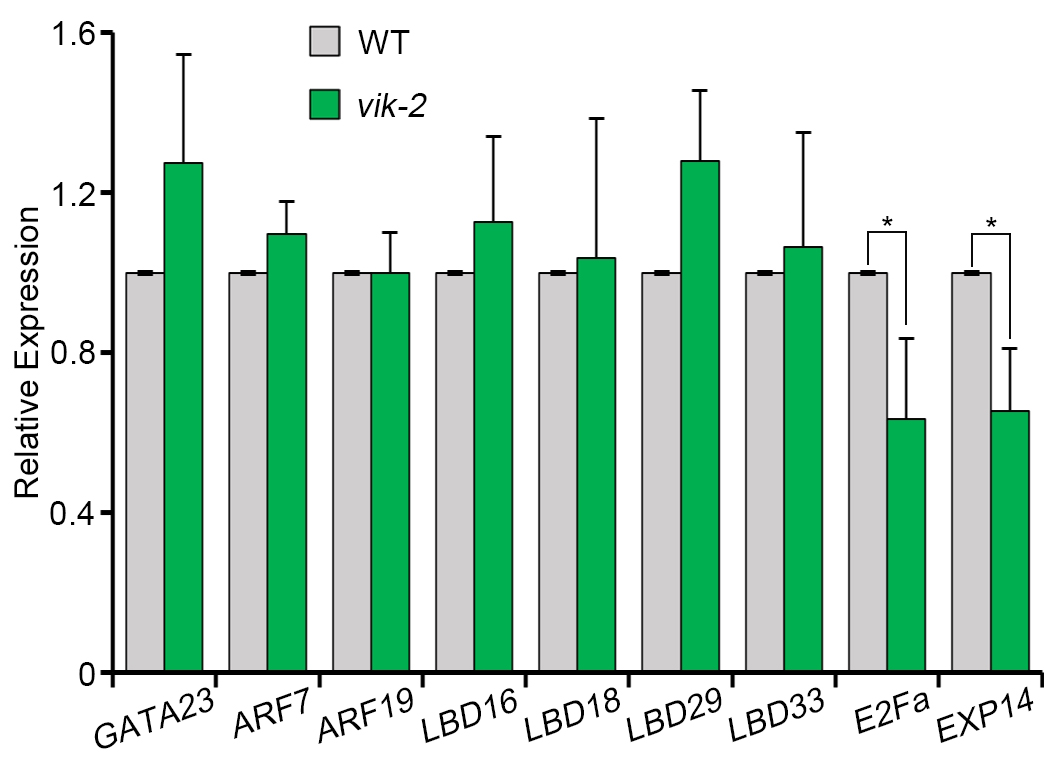
**

**Figure S17**.

Transcript level analysis of key genes involved in LR formation. The mRNA level analysis of key genes involved in LR formation in roots of *vik-2* mutant seedlings. The mRNA levels were quantified by qRT-PCR and *ACTIN2* served as an internal control. Error bars indicate SD of three biological replicates. Asterisks represent significant differences compared to WT used Student’s *t* test (**P* < 0.05).

**
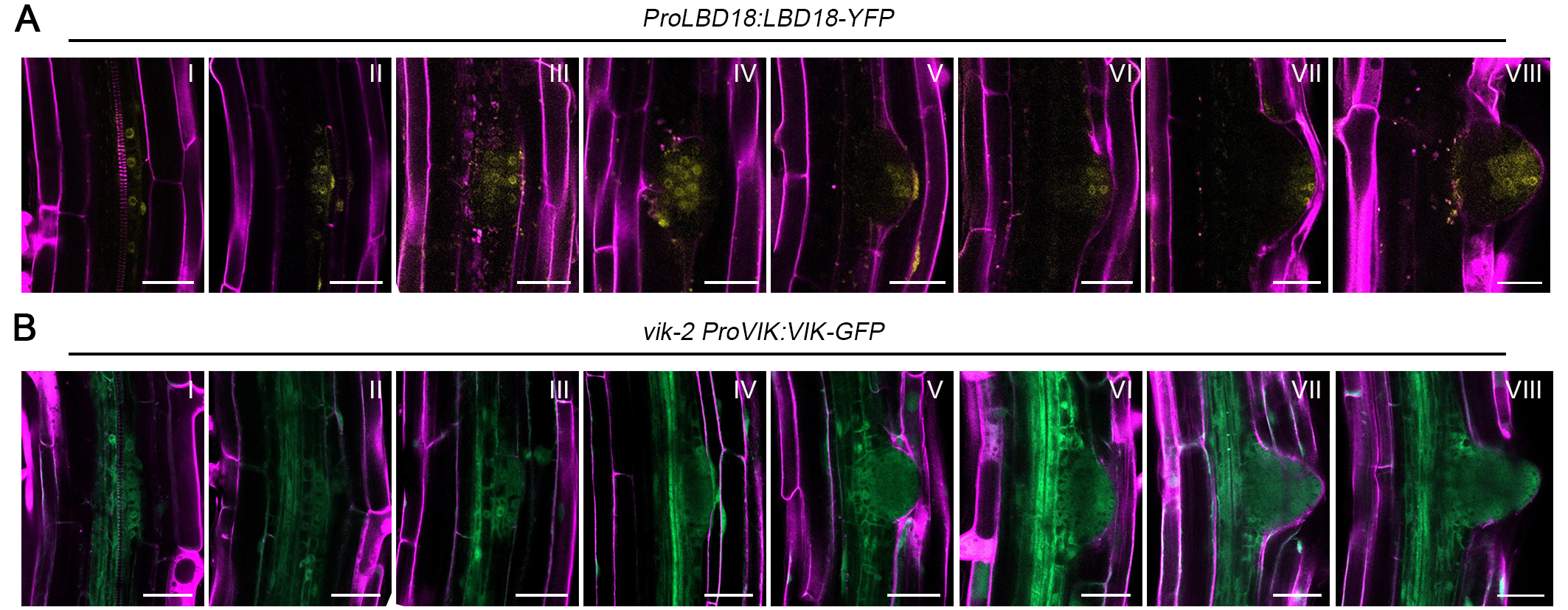
**

**Figure S18**.

The expression patterns of LBD18 and VIK in LRs. A) The expression pattern of LBD18 (yellow) in LRs from 10-day-old *ProLBD18: LBD18-YFP* transgenic plants. B) The expression pattern of VIK (green) in LRs from 10-day-old *vik-2* *ProVIK:VIK-GFP* transgenic plants. The roman numeral indicates developmental stage of LR. Yellow and green represent YFP and GFP signals, respectively. PI staining indicates cell boundary. Scale bars, 40 μm.

**
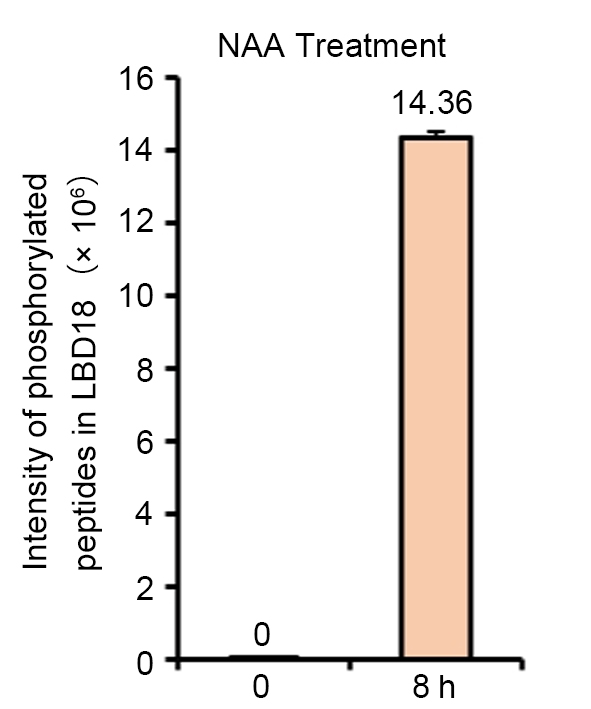
**

**Figure S19**.

The phosphorylation of LBD18 was triggered by auxin observed in phosphorylomics analysis. The phosphopeptides intensity of LBD18 from root total protein of 10-day-old WT seedlings with or without NAA treatment (10 μM NAA for 8 h).

**
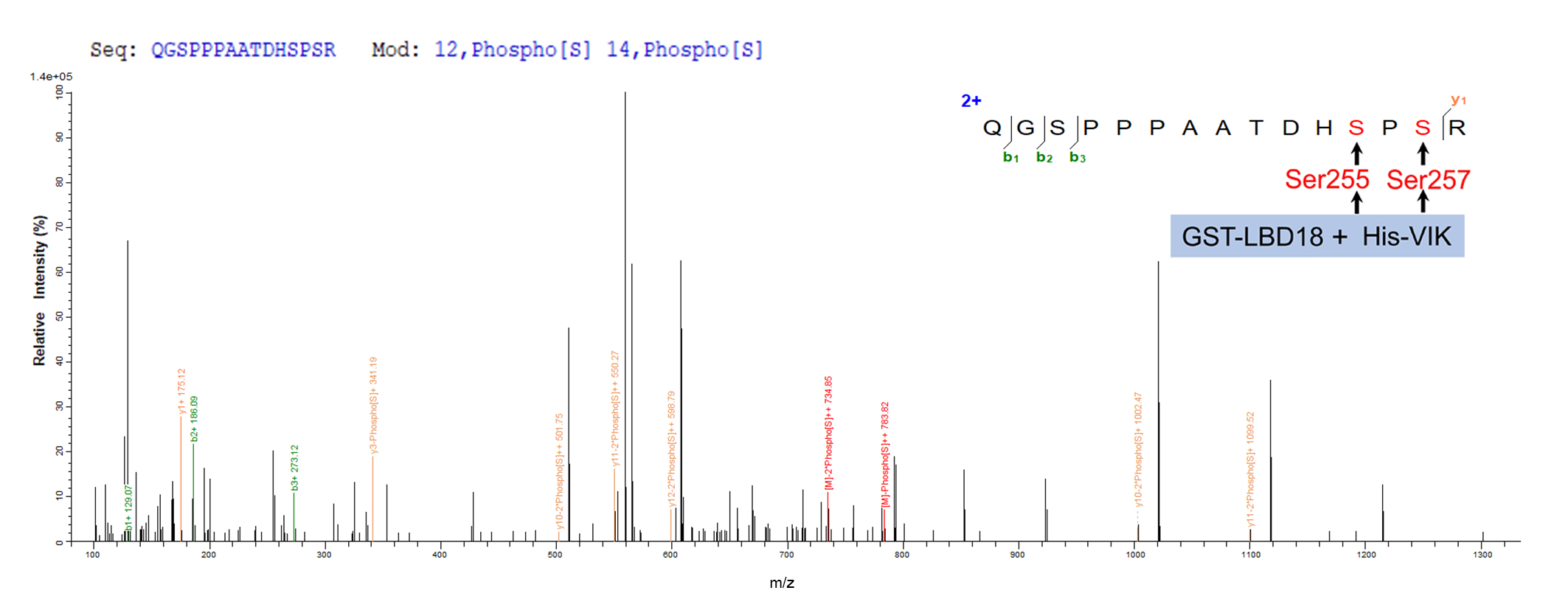
**

**Figure S20**.

The relative abundances of phosphopeptides in LBD18 detected by phosphorylation-mass spectrum assay. Recombinant GST-LBD18 was incubated with His-VIK in reaction buffer containing 10 mM ATP. The peaks marked in red were denoted phosphorylated amino acid residues Ser255 and Ser257 in a peptide fragment of LBD18 protein in phosphorylation-mass spectrum assay. The ordinate and abscissa indicate relative intensity and mass charge ratio (m/z) respectively.
