# Supplementary material for: VIK‐Mediated Auxin Signaling Regulates Lateral Root Development in Arabidopsis
Source: Adv Sci (Weinh). 2024 Jul 3;11(33):2402442. doi: 10.1002/advs.202402442 (PMC11434109; doi:10.1002/advs.202402442)
Supplement: Supplementary file 3 — Supplemental Table 2 [file ADVS-11-2402442-s003.pdf]

ADVANCED SCIENCE
Open Access

Supporting Information

for *Adv. Sci.*, DOI 10.1002/advs.202402442

VIK-Mediated Auxin Signaling Regulates Lateral Root Development in *Arabidopsis*

*Erlei Shang, Kaijing Wei, Bingsheng Lv, Xueli Zhang, Xuefeng Lin, Zhihui Ding, Junchen Leng, Huiyu Tian and Zhaojun Ding**

Table S2 Primer information.

**Vectors construction**	
**Primer Name**	**Sequence 5' to 3'**
ProVIK(pENTR-D)-F	GAAGTTTCAATGGAATCGCTAA
ProVIK(pENTR-D)-R	CTCAAAACCGATTCAGATGATTG
3'UTR-VIK(pENTR-D)-F	TACACAAAATCCATTATTTAAGCAC
3'UTR-VIK(pENTR-D)-R	AGCCAAGTCGAAGTAAGTTTTT
VIK-CDS-F	ATGAGCTCCGATTCACCGG
VIK-CDS-R	TGAAGTGAATAAGCCCCAATG
ProERF13(pENTR-D)-F	AACCACGCCTCTAGCTTTAATA
ERF13-CDS-F	ATGAGCTCATCTGATTCCGTTAATA
ERF13-CDS-R	TATCCGATTATCAGAATAAGAACAT
ProLBD18(pENTR-D)-F	GCCTGAAATGCAACCACTGTC
ProLBD18(pENTR-D)-R	GACCGGCCACCCCCCAAT
LBD18-CDS-F	ATGAGCGGTGGTGGGAACA
LBD18-CDS-R	TCATCTAGACATAGTTCGAGACGGC
MPK14-CDS-F	ATGGCGATGCTAGTTGATCCT
MPK14-CDS-R	TTAAGCTCGGGGGAGGTAATGA
TMK1-CDS-F	ATGAAGAAAAGAAGAACCTTTCTTCTA
TMK1-CDS-R	TCGTCCATCTACTGAAGTG
$TMK1^{KD}$-F	ATGAAAAAGAGGCAGAAGAGATTCTC
**Genotyping**	
LP(*vik-2*)	CAAATCCGCTGCTCATAAATC
RP(*vik-2*)	ACCATTACCATCTCCTGAGGG
LP(*vik-3*)	TATCCGGATCGTTGACTTGAC
RP(*vik-3*)	TTCCACCTATCCTGAGCATTG
**qRT-PCR**	
VIK(RT)-F	GGACATAGACCTACATTCCGTTC

VIK(RT)-R	TATGAAGTGAATAAGCCCCAAT
ARF7(RT)-F	TGCCTCTGGAACTTCTTACGG
ARF7(RT)-R	TTATCAACATTAGCTCCACCGA
ARF19(RT)-F	ATGTATCCACAGCGGGAACG
ARF19(RT)-R	AGTTACGGCTTCGAGATTACCAG
LBD16(RT)-F	AATCTTTCAGCAACACGAGCA
LBD16(RT)-R	TCTAAGAGCCAAAGCCTGAAG
LBD18(RT)-F	TCAGTCTCACCCTTGCCCTC
LBD18(RT)-R	ACGGCGACAGAAGAGGAAGA
LBD29(RT)-F	TTCTGGGACGGTTCAACACG
LBD29(RT)-R	GAAAGTGTTCAGGTGTTGTTCCA
LBD33(RT)-F	CGTTGCTCACATCTTCGCTCT
LBD33(RT)-R	CTTCCGTCGGCATTGTTCA
E2Fa(RT)-F	AACCCAGAAACTGCTATTGTG
E2Fa(RT)-R	GAGAGAAGCCAGTAGTCCGA
EXP14(RT)-F	GCCCATCTTCCTCCGTATT
EXP14(RT)-R	TAAGTACGAGGTTGAAGTATGAATG
GATA23(RT)-F	TGTGGAGAGGTGGACCAACTG
GATA23(RT)-R	GCCACCGTGTGATGATGATAA
ERF13(RT)-F	GGTTCTTGTAAGTATGAGCCG
ERF13(RT)-R	CACCGTGAAATCCAACTCC
KCS8(RT)-F	GCTTGCCTTTGAGCATTTCTG
KCS8(RT)-R	AATCTGCCAAATCCTATCTCCTT
KCS16(RT)-F	TCGCATTCGAGCATTTCTGTA
KCS16(RT)-R	CCTATCTCCTTTCGTCATCCTTC
